# Recent advances on the development of phantoms using 3D printing for imaging with CT, MRI, PET, SPECT, and ultrasound

**DOI:** 10.1002/mp.13058

**Published:** 2018-07-24

**Authors:** Valeria Filippou, Charalampos Tsoumpas

**Affiliations:** ^1^ Institute of Medical and Biological Engineering Faculty of Mechanical Engineering University of Leeds Leeds LS2 9JT West Yorkshire UK; ^2^ Department of Biomedical Imaging Science School of Medicine University of Leeds Leeds LS2 9NL West Yorkshire UK

**Keywords:** 3D printing, CT, image quality, mammography, MR, PET, phantoms, SPECT, US

## Abstract

**Purpose:**

Printing technology, capable of producing three‐dimensional (3D) objects, has evolved in recent years and provides potential for developing reproducible and sophisticated physical phantoms. 3D printing technology can help rapidly develop relatively low cost phantoms with appropriate complexities, which are useful in imaging or dosimetry measurements. The need for more realistic phantoms is emerging since imaging systems are now capable of acquiring multimodal and multiparametric data. This review addresses three main questions about the 3D printers currently in use, and their produced materials. The first question investigates whether the resolution of 3D printers is sufficient for existing imaging technologies. The second question explores if the materials of 3D‐printed phantoms can produce realistic images representing various tissues and organs as taken by different imaging modalities such as computer tomography (CT), positron emission tomography (PET), single‐photon emission computed tomography (SPECT), magnetic resonance imaging (MRI), ultrasound (US), and mammography. The emergence of multimodal imaging increases the need for phantoms that can be scanned using different imaging modalities. The third question probes the feasibility and easiness of “printing” radioactive or nonradioactive solutions during the printing process.

**Methods:**

A systematic review of medical imaging studies published after January 2013 is performed using strict inclusion criteria. The databases used were Scopus and Web of Knowledge with specific search terms. In total, 139 papers were identified; however, only 50 were classified as relevant for this paper. In this review, following an appropriate introduction and literature research strategy, all 50 articles are presented in detail. A summary of tables and example figures of the most recent advances in 3D printing for the purposes of phantoms across different imaging modalities are provided.

**Results:**

All 50 studies printed and scanned phantoms in either CT, PET, SPECT, mammography, MRI, and US—or a combination of those modalities. According to the literature, different parameters were evaluated depending on the imaging modality used. Almost all papers evaluated more than two parameters, with the most common being Hounsfield units, density, attenuation and speed of sound.

**Conclusions:**

The development of this field is rapidly evolving and becoming more refined. There is potential to reach the ultimate goal of using 3D phantoms to get feedback on imaging scanners and reconstruction algorithms more regularly. Although the development of imaging phantoms is evident, there are still some limitations to address: One of which is printing accuracy, due to the printer properties. Another limitation is the materials available to print: There are not enough materials to mimic all the tissue properties. For example, one material can mimic one property—such as the density of real tissue—but not any other property, like speed of sound or attenuation.

## 1 Introduction

Imaging technology is traditionally used as a noninvasive tool to map the anatomy and/or the function of the human body, as well as to detect and localize the process of a disease. Nowadays, several new medical imaging methods and techniques have been developed to offer information about the function, physiology, and metabolism of an organ. Medical images offer accurate diagnoses, enhanced visualization and effective individual treatments for a range of diseases.

There are three main types of tomographic medical imaging: imaging using x rays, molecular radionuclide imaging, and nonionizing imaging. Each of these consists of several imaging modalities. This review focuses on CT, mammography, PET, SPECT, MRI, and US. To clinically validate these systems in different circumstances, several tests are undertaken using physical phantoms. There are several types of available phantoms which reflect the numerous imaging tasks, such as geometrical accuracy, dose algorithm accuracy, image quality, machine and patient quality assurance, irradiation techniques, and calibrations of measurements to required physical quantities.^1^

Traditional mold phantoms are used by several researchers and radiologists to validate imaging systems.^2^, ^3^, ^4^, ^5^, ^6^ However, these phantoms have limitations: for example, the complex geometry and structure of the human body cannot be completely replicated, and they are relatively expensive. Some mold phantoms may not have realistic structures, and therefore the evaluation of imaging methods and systems is often limited with their use. The solution to overcome these problems may be offered by 3D printing technology, which has become more accessible, versatile, and accurate.^7^ This review focuses primarily on these types of phantoms. To develop an anthropomorphic phantom, computer‐aided design (CAD) software can be used without prior information, or an image can be extracted from an imaging scanner.^8^, ^9^ This often provides the opportunity to clinicians, physicists, technologists and radiographers to develop much more complex structures of a given phantom.^10^ The cost of the material constitutes the main expense when 3D printing. Baba et al.^11^ provides useful data on the expenses associated with 3D‐printed material. Another benefit of using 3D printing technology is the economic production of low batch sizes allowed by the usage of a common material independent of the end geometry.^12^ This is significant in terms of the development of medical applications for specific patients. 3D printing can be achieved either directly (where the phantom is printed) or indirectly (where the casting mold is printed and other materials are used to build the phantom).^11^, ^13^, ^14^, ^15^, ^16^, ^17^, ^18^, ^19^, ^20^, ^21^, ^22^

This article investigates the quality of the image produced when a 3D‐printed phantom is scanned by one or more of the imaging modalities introduced earlier. Image quality can be assessed either quantitatively or qualitatively.^23^, ^24^ The former is achieved by measuring different properties, such as the attenuation coefficient, density, geometry and Hounsfield Units (HU) of the phantom—all of which can be compared against the expected values.^25^ The 3D‐printed phantoms are firstly categorized based on the imaging modality of use, and then according to the type of tests. The phantoms examined are primarily anthropomorphic (meaning that they represent body parts), however, a few geometrical phantoms are included as well. Finally, the anthropomorphic phantoms are further classified in the organ type that they represent: for example, skeletal, muscular, cardiovascular, digestive, endocrine, nervous, respiratory, urinary, and reproductive.

*Hypothesis and questions*:

A single 3D‐printed phantom can be used for imaging in different imaging acquisitions and modalities to help improve imaging systems for more realistic and accurate experiments.

The following questions are addressed:

1. Is the resolution of current 3D printers sufficient to develop a detailed and realistic phantom?
2. Do current materials offer the range of imaging densities of different tissues?
3. Is it feasible and practical to include a radiotracer or a nonradioactive solution inside the printing materials?

## 2 Materials and methods

**Figure 1:**
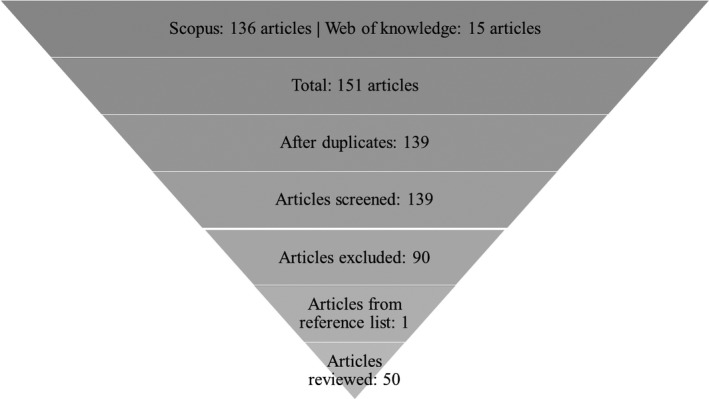
Search strategy of the review article.

A systematic review was conducted of articles related to CT, MRI, PET, SPECT, US, and/or mammography published after January 2013, and to phantoms which were developed for usage in those imaging systems. On the 23rd of April 2018, a literature search to identify articles for this review paper was carried out through the following search engines: *Scopus* and *Web of Knowledge*. The terms “3D print*”, “three dimensional print*”, “3 dimensional*”, “3‐D print*”, “three‐dimensional print*”, “3‐dimensional”, “additive”, “rapid prototyping”, “phantom*”, “physical model*”, “CT”, “MRI”, “MR”, “PET”, “SPECT”, “ultrasound”, “mammogra*” were used in the search fields of those databases. In Scopus, these terms were used to search specifically in the title, abstract, and keywords of the article. Web of Knowledge allows users to search only the titles of articles, therefore, a big difference in the results’ numbers in each database is observed, as illustrated in Fig. 1. The terms used were related to the inclusion criteria of the review and the manufacturing method of the phantoms. The studies were excluded if: (1) The phantoms were not developed using 3D printing. (2) The studies were not related to imaging with CT, MRI, PET, SPECT, or Ultrasound. (3) The articles were published before 2013. (4) The articles were not published in peer‐reviewed journals. (5) They were published in journals unrelated to the scope of using 3D‐printed phantoms for medical imaging (such as *Surgical Endoscopy & Other Interventional Techniques*).

Following that process #bib139 articles were identified in both databases, and then any duplicates were excluded. The remaining titles and abstracts were manually screened to select only the most relevant articles for this review. The remaining articles were excluded if: (a) an actual phantom was not 3D‐printed, (b) the phantom was not 3D‐printed but just designed, (c) the results of the articles were not related to our hypothesis, (d) the phantoms were not imaged by any imaging modality considered for this review, (e) older studies were followed by subsequent publications. In addition, the references of the chosen articles were further screened to select any other relevant articles. Figure 1 shows the process followed up for the selection of the papers of this review.

**Figure 2:**
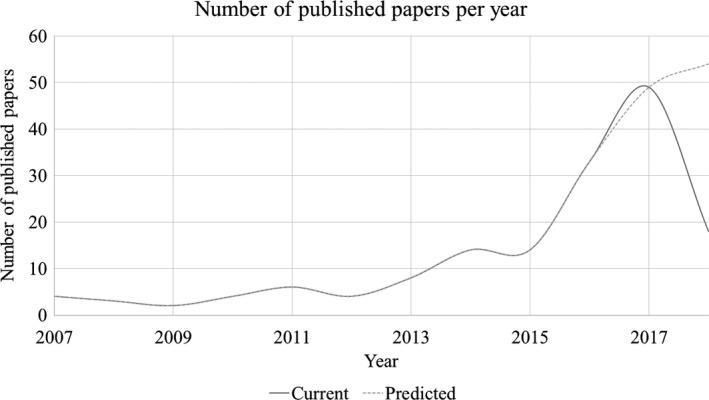
Research articles published per year between 2007 and 2018 in Scopus database.

**Figure 3:**
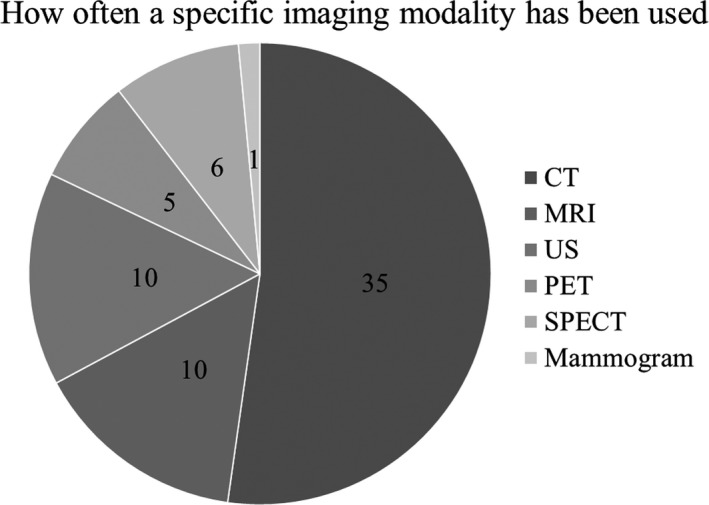
Number of research articles that used these imaging modalities to scan the 3D printed phantoms (starting with CT clockwise).

Figure 2 demonstrates the number of research articles that were published between 2007 and 2018 in Scopus. In total, 162 articles were identified, whereas there were 139 articles published between 2013 and 2018. These numbers clearly demonstrate that there is an upward trend of published research articles related to 3D‐printed phantoms. Figure 3 shows the number of research articles associated with each imaging modality. Most of the articles (12) discussed in this review were published in *Medical Physics*. Other journals with such published articles were *Magnetic Resonance in Medicine* with four articles and the *European Journal of Nuclear Medicine & Molecular Imaging Physics*, and *Physica Medica*, with three articles each.

## 3 Results

As shown in Fig. 1, 50 studies were selected to be reviewed for the purposes of this manuscript. Table 1 summarizes the data identified in terms of phantoms, printers and imaging systems in all 50 articles. Table 2 shows the properties of the printers that were used by the selected articles. Table S1 demonstrates the measurements undertaken by each group to measure the accuracy of the 3D‐printed phantoms, and whether the measurements were qualitative or quantitative.

**Table 1:** Information of each article regarding the phantoms, medical imaging scanners and their activity (if present), Direct: 3D printed phantom vs Indirect: 3D printed mold of the phantom

First author	Year	Direct vs indirect	Phantom appearance (phantom category)	Phantom material	Radiotracer (if used)	Imaging modality
Geometrical phantoms						
Madamesila^60^	2016	Direct	Lung‐cylinder (geometrical)	High impact polystyrene	–	CT
Solomon^37^	2016	Direct	4 cylinders with 20 low contrast spherical signals (geometrical)	TangoPlus, VeroWhite	–	CT
Dancewicz^61^	2017	Direct	Filaments (geometrical)	ABS, PLA, Photoluminescent PLA, Woodfill, Bronzefill, Copperfill, Standard photopolymer resin, Flexible photopolymer resin (different combinations)	–	CT
Seoung^30^	2017	Direct	Cylinder (geometrical)	ABS, PLA	–	CT
Shin^62^	2017	Direct	Circular (geometrical)	PLA, ABS, polyethylene terephthalate (PET), TPU, high impact polystyrene (HIPS), PVA, Nylon	–	CT
Ceh^29^	2017	Direct	Filaments and head (geometrical, nervous)	ABS (1.04), ABS‐Bi 1 (1.20), ABS‐Bi 2 (1.30), ABS‐Bi 3 (1.60), ABS‐Bi 4 (1.90), ABS‐Bi 5 (2.20), ABS‐Bi 6 (2.50), GMASS (2.7)	–	CT
Torso phantoms						
Javan^38^	2016	Direct and indirect	Spine (skeletal)	Gypsum, rubber‐like, Ecoflex 00‐50, polyamide, gelatin and calcium chloride	–	CT
Kadoya^22^	2017	Direct and indirect	Pelvis (uterus, bladder), (skeletal, urinary)	VeroCyan, silicone, water	–	CT
Lin^39^	2017	Direct	Trabecular bone (skeletal)	–	–	CT
Oh^50^	2017	Direct	Spine (skeletal)	UVAP, plastic powder, titanium, agar liquid	–	CT
Shen^27^	2017	Direct and indirect	Skeleton, spine nerve, colon, kidney‐bladder, other tissue (skeletal, urinary)	Silica gel, ABS, plasticine	–	CT
Craft^49^	2017	Direct	Chest, mastectomy (skeletal, female reproductive system)	PLA	–	CT
Lee^26^	2016	Indirect	3D printed RANDO (whole body)	PDMS, mixture of wax and tungsten powder	–	CT
Leng^63^	2016	Direct	Liver, brain (digestive, nervous)	TangoBlack +, FLX 9895, RGD 8530, RGD 8505	–	CT
Vessel phantoms						
Toepker^28^	2013	Direct	Vessels, stenotic lesions (cardiovascular)	FullCure 720, TangoBlack	–	CT
Hamedani^54^	2018	Direct	(a) cylinder, (b) artery tree, (c) pelvis, (d) iliac artery	ABS, Barium sulfate	–	CT
Hazelaar^55^	2018	Direct and indirect	Thorax with lung cancer	Gypsum, nylon, silicone, PMMA	–	CT
Joemai^64^	2017	Direct	Chest with lung vessels	VisiJet EX200, PMMA	–	CT
Kamomae^51^	2017	Direct	Head	PLA	–	CT
O'Dell^31^	2017	Direct	Arterial tree	ABS	–	CT
Geometrical phantoms						
Yoshimaru^65^	2014	Direct	Rectangle (almost) (geometrical)	Fullcure 720 polymer	–	MRI
Head phantoms						
Kasten^58^	2016	Direct	Brain (nervous)	ABS coated with epoxy resin, corn oil, N‐acetyl‐L‐aspartic acid, creatine, choline chloride, Na‐L‐LACTATE	–	MRI
Wood^36^	2017	Direct and indirect	Head phantom (brain, brainstem, air cavities, CSF, cerebellum, eyes, muscle, fat, bone, skin) (skeletal, nervous)	SLA resin DSM Somos WaterShed XC11122, distilled water, sodium chloride, denatured ethanol	–	MRI
Saotome^56^	2017	Direct	Brain (skeletal, nervous)	FullCure 810, agarose gel	–	MRI
Bone phantoms						
Rai^66^	2018	Direct	Cortical bone (skull, tibia)	Photopolymer resin (bone), doped water, undoped water, Gd‐DTPA (skull), vegetable oil (bone marrow‐tibia), gelatine (soft tissue—tibia)	–	MRI
Torso phantoms						
Adusumilli^45^	2014	Indirect	Shoulder (skeletal)	DureForm PA nylon 12‐based, gelatin, psyllium husk powder, chlorhexidine	–	US
Bücking^33^	2017	Direct	Ribcage, liver, right lung (skeletal, digestive, respiratory)	“Enhanced polymax” PLA	–	US
Geometrical phantom						
Fuzesi^47^	2017	Direct	Rectangle (geometrical)	ABS, PLA, thermoplastic polyurethane (TPU)	–	US
Nikitichev^67^	2016	Direct	Rectangle (geometrical)	(a) VeroWhite Plus, VeroBlue, (b) PolyMax	–	US
Vessel phantoms						
Lai^68^	2013	Direct	Vessels (cardiovascular)	FullCure 930 and FullCure 705 and agar‐based mixture (water, agar, glycerol, silicon dioxide, potassium sorbate preservatives)	–	US
Morais^46^	2017	Indirect	Atrial (cardiovascular)	Silicone, PVA‐C	–	US
Maneas^44^	2018	Indirect	Nerve and vessel (not printed)/heart atrium/placenta	Gel wax, paraffin wax, glass spheres	–	US
Geometrical phantoms						
In^69^	2017	Direct	Cylinders mimicking liver (geometrical)	Silicone gel, UV electro225 catalyst, UV LSR catalyst	–	CT, MRI, US
Alssabbagh^40^	2017	Direct	Cubes, thyroid (geometrical, endocrine)	PLA, ABS, Polyethylene terephthalate glycol (PETG), thermoplastic elastomers (TPE), polyamide (PA)	^99m^Tc	CT, Scintigraphy
Head phantom						
Gallas^20^	2015	Direct	Head (skeletal and nervous)	Epoxy resin (outer phantom and soft bone), K_2_HPO_4_ in water (bone compartment), agarose gel and water (brain), water (ventricle), BANG 3‐Pro gel (tumor)	–	CT, MRI
Torso phantoms						
Mitsouras^52^	2017	Direct	C6‐C8 vertebra, tumor (skeletal)	RGD‐525 (tested 17 materials, for more information refer to the paper)	–	CT, MRI
Niebuhr^42^	2016	Direct, indirect and traditional	Pelvis (skeletal)	Pelvis case (PMMA), hollow bone (VeroClear), hollow organ shells (neukasil), soft‐tissue (agarose gel + Ga‐based contrast agent + NaCl + NaOH + NaF), fats and inner bone (vegetable oil, animal fats and vaseline, K_2_HPO_4_), gypsum	–	CT, MRI, teletherapy
Laing^53^	2018	Direct and indirect	Heart and valve model	PLA, Silicone, PVA‐C	–	CT, US
Adams^21^	2016	Direct and indirect	Kidney (urinary)	Silicone, agarose, Polydimethylsiloxane (PDMS)	–	CT, US, Endoscopy
Geometrical phantoms						
Wollenweber^59^	2016	Direct	Cylinder (geometrical)	Acrylic spheres in fillable tank plus nylon features	^18^F and 400 ml water and 1 drop of surfactant (phantom 1)	PET, CT
Gallivanone^70^	2016	Indirect	Irregular and nonhomogeneous lesions (geometrical)	Radioactive aliginate gel	^18^F‐FDG with water	PET/CT
Cerviño^71^	2017	Direct	Cylinder (geometrical)	ABS—P430	^18^F‐FDG, H_2_O	PET/CT
Bieniosek^10^	2015	Direct	Cylinder (geometrical)	VisiJet M3 Crystal plastic	^18^F (PET/CT and PET/MRI)	CT, PET/CT, PET/MRI
Torso phantoms						
Gear^32^	2016	Direct	Cubic samples, liver, lungs, abdominal trunk, lesions (geometrical, digestive, respiratory)	VeroWhite Plus FullCure 835, TangoBlack Plus FullCure 980 Shore 27a, VeroClear FullCure 810	^90^Y SPECT/CT and PET/CT, ^99m^Tc SPECT/CT	PET/CT, SPECT/CT
Robinson^34^	2016	Direct	Spleen, kidney, pancreas and liver (digestive and urinary)	ABS plastic	^99m^Tc, ^177^Lu	SPECT
Woliner van der Weg^72^	2016	Direct	Pancreas and kidney (digestive and urinary)	VeroClear RGD 810	^111^In‐exendin	SPECT/CT
Tran‐Gia^35^	2018	Direct	Kidney (urinary)	PLA	^177^Lu	SPECT/CT
Head phantoms						
Negus^41^	2016	Direct	Brain (nervous)	Polyactide (PLA)	^99m^Tc solution in ink—printed on paper	SPECT
Endocrine phantoms						
Alqahtani^73^	2017	Direct	Thyroid gland (endocrine)	ABS	^99m^Tc	SPECT/CT
Reproductive system phantoms						
Kiarashi^48^	2015	Direct	Breast (female reproductive system)	TangoGray, VeroWhite, TangoPlus	–	Mammogram

**Table 2:** Properties of the 3D printers

Printer brand	First authors	Printer model	Vertical resolution (layer thickness, Z) (μm)	Horizontal resolution (XY resolution) (μm)	Accuracy (μm)	Build volume (mm)
PolyJet/MultiJet/InkJet technology						
Stratasys	Gear^32^	Connex3 series	16	42 × 42	20–85 (features <50 mm) #bib200 (full model size)	–
	Kiarashi,^48^ Kadoya,^22^ Mitsouras^52^	Objet 500 Connex				490 × 390 × 200
	Yoshimaru,^65^ Solomon,^37^ Leng^63^	Objet 350 Connex				342 × 342 × 200
	Gear^32^	Objet Eden 500V	16	42 × 42	20–85 (features <50 mm) #bib200 (full model size)	500 × 400 × 200
	Niebuhr,^42^ Nikitichev^67^	Objet 30 Pro	28	42 × 42	100	294 × 192 × 148.6
	Woliner van der Weg^72^	Objet Eden250	16	42 × 42	100	255 × 252 × 200
	Adams^21^	Objet 260 Connex 3	16	42 × 42	20–85 (features <50 mm) #bib200 (full model size)	255 × 252 × 200
	Toepker,^28^ Wood,^36^ Saotome^56^	Eden 350				
Object geometries	Carton^74^	Objet Eden 500V	16	42 × 42	20–85 (features <50 mm) #bib200 (full model size)	500 × 400 × 200
3D systems	Bieniosek,^10^ Bieniosek,^10^ Mooney,^75^ Joemai^64^	ProJet HD3500	16–32	42 × 42	–	298 × 185 × 203
	Oh^50^	Projet 5000	29	34 × 34	–	‐
Zcorp (now 3D systems)	Hazelaar^55^	Zcorp 650	89–102	42 × 47	–	254 × 381 × 203
Fused deposition modeling						
Stratasys	Kasten^58^	Fortus 250mc	330 #bib254 #bib178	–	±241 (geometry dependent)	254 × 254 × 305
	Robinson,^34^ O'Dell^31^	Dimension Elite	254 #bib178	–	–	200 × 200 × 300
	Seoung^30^	Fortus 400mc	330 #bib254 #bib178 #bib127	–	±127	406 × 355 × 406
	Cerviño^71^	uPrint SE Plus	254–330	–	–	203 × 203 × 152
3D systems	Dancewicz^61^	3D touch	125	–	±1% of object dimension or ±200 μm (0.008”/200 μm) whichever greater (XY), ±half processed (Z) resolution	Single head 275 × 230 × 185
MakerBot Industries	Dancewicz,^61^ Ceh^29^	MakerBot Replicator 2	–	72	11 (XY) #bib2.5 (Z)	285 × 153 × 155
	Lee^26^	MakerBot Z18				
RepRapPro	Negus^41^	RepRapPro Mendel	300	–	100	200 × 200 × 140
Ultimaker	Bücking,^33^ Nikitichev,^67^ Morais,^46^ Maneas^44^	Ultimaker 2	250 nozzle: 60–150	–	12.5 #bib12.5, 5 XYZ	223 × 223 × 205
			400 nozzle: 20–200			
			600 nozzle: 20–400			
			800 nozzle: 20–600			
	Laing^53^	Ultimaker 3	250 nozzle: 60–150	–	12.5 #bib12.5, 5 XYZ	215 × 215 × 200
			400 nozzle: 20–200			
			800 nozzle: 20–600			
Aleph objects	Dancewicz,^61^ Shin,^62^ Hamedani^54^	Lulzbot Taz 5 desktop	350 nozzle: 75–350	–	–	290 × 275 × 250
			500 nozzle: 75–500			
	Hamedani^54^	Lulzbot Taz 6	500 nozzle: 50–500			280 × 280 × 250
re:3D	Craft^49^	Gigabot 2.0	100–300	4		–
Dong Guan Pioneer Trading Co	Alssabbagh^40^	–	5	20		–
	Kamomae^51^	Ninjabot FDM‐200	50	–	–	200 × 200 × 200
Renkforce	Gallivanone,^70^ Tran‐Gia^35^	RF1000				
Stereolithography (SLA) ‐ (STL, stereolithography file format)						
Prismlab	Rai^66^	Prismlab RP400	100 #bib50	100 #bib67 #bib50	–	384 × 216 × 380

Conversion of dpi to μm → $\frac{25,400\;\mu m}{x\;dpi}=y\;\mu m$, where 25,400 μm is 1 inch—all resolution conversions were rounded to the nearest integer number.

### 3.A. Characterization of 3D‐printed phantom spatial accuracy

The resolution of the 3D printers is significant in the produced images from CT, PET, SPECT, US, MRI, and mammography for the visualization of the phantom. The resolution is expressed in dots per inch (dpi) or micrometers (μm) and assessed by comparing the dimensions of the produced physical phantom with the original dimensions provided to the printer. Among the papers used, 10 have assessed the resolution or accuracy of the printer by quantitative (numerical) comparison,^26^, ^27^, ^28^, ^29^, ^30^, ^31^, ^32^, ^33^, ^34^, ^35^ 10 by qualitative (figural) comparison,^22^, ^36^, ^37^, ^38^, ^39^, ^40^, ^41^, ^42^, ^43^, ^44^ and 14 by both quantitative and qualitative comparison.^10^, ^21^, ^45^, ^46^, ^47^, ^48^, ^49^, ^50^, ^51^, ^52^, ^53^, ^54^, ^55^, ^56^ Sixteen of the research articles do not include a verification of the printers’ resolutions.

#### 3.A.1. Computed tomography

Lee et al.^26^ developed an identical phantom to the Alderson RANDO phantom in eight horizontal slices that were combined to form the entire phantom. The Alderson RANDO phantom is an anthropomorphic phantom that is able to represent both genders. It is a torso that includes the head, neck, chest, pelvis, and for the female phantom, a breast attachment is available. To measure the accuracy of the 3D‐printed phantom, the planned and actual thickness of the slices were measured. The average error of the eight slices was 0.48 ± 0.27 cm. In addition, the fabrication error was measured, with an average result of 0.16 ± 0.15 mm. CT images of the RANDO phantom and the 3D‐printed model were acquired and found to be comparable. Similarly, Craft et al.^49^ developed a mastectomy chest phantom in 11 vertical slices that formed the entire phantom when combined. To verify whether there was an error between the planned and actual slices, their thickness was measured. There was a slice error between 0.44 and 0.60 mm, with an average of 0.52 mm. It was noticed that the slice error decreased as the slice extended further from the printing bed surface due to warping. The bottom, middle, and top slice errors were measured as 0.76 #bib0.51, and 0.29 mm, respectively. The volumetric error was measured as well, with an average error of 1.37%. Last, the CT images of the phantom and patient were visually compared, providing high agreement. However, the only disagreement between them was the lungs, since the unsupported nodules were trimmed off due to 3D printer limitations.

Oh et al.^50^ and Kamomae et al.^51^ used dice similarity coefficient (DSC), which is a statistical validation metric, to compare whether the physical phantom's dimensions are consistent with the patient's model. The former author compared the volume of the external body, spine and metallic fixation screw (MFC) with the data from the real CT image of the patient. The results showed high DSC in each individual model, which were 0.98 #bib0.91, and 0.89, respectively. In addition, the volume percentage difference of the external body, spine and MFC of the 3D model and the patient was measured, resulting in 4.1%, 6.4%, and 10% error, respectively. Even though numerical differences were identified, the CT images of the patient and the phantom matched visually. Kamomae et al.^51^ also developed a head phantom. The measured difference, in terms of the shape of the head was no more than 1 mm. However, a maximum difference of 2 mm was measured on the bottom of the head phantom. The DSC was 0.974, which demonstrates high similarity.

Another author, Shen et al.^27^ developed multiple phantoms, including a skeleton, spine nerves, a colon, a kidney‐bladder, and other soft tissues. Fidelity maps were used to identify the geometrical accuracy of each individual phantom and the according patient. This method was used before and after assembling the individual phantoms, and the fidelity maps demonstrated errors before and after assembly: less than 0.5 mm and less than 1.5 mm, respectively.

In addition, Mitsouras et al.^52^ checked the differences between the cervical spine phantom and the original data from CT and MRI, using both CT and MRI scanners. The results demonstrated an average difference of 0.13 mm and 0.62 mm for CT and MRI, respectively. The difference in the model accuracy when using an MRI in comparison to CT is much larger, however, it is still less than two‐thirds of the imaging resolution used.

Furthermore, Toepker et al.^28^ developed six phantoms to represent coronary arteries with stenosis. The results demonstrated that smaller areas and diameters had greater degrees of error in comparison to larger areas and diameters. For example, a 0.20 mm^2^ area with a 0.5 mm diameter had a difference error of 664% from its true size, while a 12.57 mm^2^ area with a 4 mm diameter had a difference error of 17%.^28^

Furthermore, Ceh et al.^29^ compared several anatomical features, such as the zygomatic bone, the middle turbinate bone, the upper mandible and lower maxilla, between the CT scan of a patient and the phantom measurements in terms of width. The average percentage difference of the anatomical features was 1.71%.

A different method was used by Adams et al.^21^ to determine the differences between the kidney phantom and the original model. The method used involved the 3D triangular mesh editing software “CloudCompare”. It demonstrated a 2 mm distance error between the original data and the 3D‐printed phantom, with a mean error of 0.6 mm. Similarly, Laing et al.^53^ used this software to measure the difference between the 3D‐printed cardiac phantom and the original patient model. A histogram and a color map were produced showing the Euclidean offset distance between the two images. The average distance error was 0.98 mm.

Seoung et al.^30^ developed a cylinder with four spots and compared the ideal (5 mm and 10 mm) and measured thickness, resulting in errors of 8% and 9%, respectively. Note that the percentage differences mentioned in our manuscript were either noted explicitly by the authors or calculated using percentage difference formula: *(original value−new value)/original value*.

Hamedani et al.^54^ developed five filaments using different combinations of Acrylonitrile butadiene styrene (ABS), barium sulfate and mineral oil. Four phantoms—cylinder, artery tree, pelvis, and iliac artery tree—were 3D‐printed using the filaments. Each phantom was used to assess different parameters. For example, the artery tree phantom was developed to assess the printer's accuracy when producing small‐diameter cylinders. The specified value of each diameter was known, and a caliper was used to measure the diameter of the 3D‐printed artery at different locations on the phantom. The size of the diameters checked was 1 #bib2, and 4 mm with errors of −7.1%, −4.45%, −2.18%, respectively.

Hazelaar et al.^55^ compared the STL models to identify any differences between the phantom and the models used to print the phantom were measured using a function called “local best‐fit”. The differences in the soft tissue, the right and left scapula, the ribcage, the lung, and the tumor were measured, with the smallest difference found in the tumor (−0.03 ± 0.76 mm) and the largest in the soft tissue (−0.75 ± 0.86 mm).

O'Dell et al.^31^ developed a 3D arterial tree phantom to validate a mathematical method used to measures the size of vessels. The physical model was consisted of 74 branches; however, only 64 branches were measurable due to the phantom design. Each branch was manually measured using a digital caliper to identify any geometric errors. The manual measurements were compared with those from the mathematical method. The standard deviation of the difference between these two methods was 0.074 mm, which demonstrates excellent agreement for all the vessel sizes.

#### 3.A.2. Magnetic resonance imaging

Saotome et al.^56^ developed a brain phantom. A different statistical method was used to measure the agreement between the patient and phantom MRI images in comparison to the other studies. Pearson's correlation coefficient of the intensity signal of the two images was measured giving *R* ^2^ as 0.955, which demonstrates good agreement. Also, the MRI images captured were visually similar.

Gear et al.^32^ developed an abdominal phantom, consisting of liver, lungs, trunk, and several lesions, for validating quantitative SIRT, the phantom parts were compared to the original MRI data of the patient. The volume difference between the liver in the printed phantom and the liver in the original MRI data had a difference of 9.6%. The longest dimension had a difference of 1.4% and the shortest dimension had practically no difference. Also, the difference between the trunk phantom and the original patient data were 2.3% in the anterior/posterior dimensions and 0.9% in the left/right dimensions.

#### 3.A.3. Ultrasound

Adusumilli et al.^45^ indirectly developed a shoulder model, and to measure the accuracy of the model, the acromiohumeral distance of the printed phantom and the US digital model was measured. Median, interquartile range and coefficient of variation were measured using calipers and sonography. All the results demonstrated excellent reliability.

Morais et al.^46^ developed two atrial phantoms with different materials, namely PVA‐C and silicone. The phantoms were scanned using US before being compared with the ideal model. Volume, point‐to‐surface error, dice and hausdorff were the parameters that were used to compare the models of left and right atrium, while only the volume was used to compare the printed models with the ideal one. The left atrium results demonstrated error difference of 12.3% between the ideal and silicone models, as well as 12.8% between the ideal and the PVA‐C models. The right atrium results demonstrated an error difference of 18.3% between the ideal and the silicon models, as well as 17.2% between the ideal and the PVA‐C models. US images of the inter‐atrial septal wall were also taken, along with images of left and right atrium.

Bücking et al.^33^ calculated the percentage difference of the phantom ribs, liver, and right lung, and the *in silico* models in terms of different features such as spinal depth, total liver height, bronchus length. The average error between the phantoms and the *in silico* models was 0.78% #bib1.3%, and 2.53% for the ribs, liver, and right lung, respectively.

Fuzesi et al.^47^ developed three rectangular phantoms made of different materials such as ABS, PLA, and photopolymer. The first two were printed using fused deposition modeling (FDM) technology and the last one using digital light processing (DLP) printing. The FDM printer printed the filaments for both materials with a 12% error. On the other hand, DLP printed the filaments at their correct position 100% correctly. Although FDM created position errors, the printing resolution was better than that of DLP. The former was able to print the filaments closely to the ideal set value, but DLP printed the filaments longer in the axial direction by 0.5 mm.^47^

#### 3.A.4. Radionuclide imaging

Bioniosek et al.^10^ compared the rod diameters of the 3D‐printed cylindrical phantom with its identical commercial one using both PET and CT systems, resulting in differences between 0.07% and 4.63%. The resultant images of the two phantoms were almost identical.^10^

Robinson et al.^34^ developed phantoms consisting of liver, spleen, kidneys (adult, age 5, and age 10), and pancreas. The volume parameter was used to compare the digital model with the 3D‐printed model. The percentage error difference was 13.8% for the liver #bib10.94% for the spleen, 17.92% for the adult kidney, 23.96% for the age 5 kidney, 17.05% for the age 10 kidney, and 23.22% for the pancreas.

Tran‐Gia (2018)^35^ developed a kidney phantom to compare different partial volume techniques based on geometries with a spherical and an ellipsoid commercial phantoms. A similar 3D‐printed phantom was developed as Tran‐Gia (2016),^57^ including a medulla part inside the cortex of the phantom. To compare the geometries, geometric recovery coefficients were calculated. The differences between sphere/ellipsoid, sphere/cortex, and ellipsoid/cortex were estimated. The sphere and ellipsoid had a difference of 0.7%, however, the sphere and the cortex had a difference of 31.7%.

#### 3.A.5. Mammography

Kiarashi et al.^48^ used a mammogram to image the phantom and reported thickness differences in the breast phantom of less than 0.05 mm from the nominal value.

#### 3.A.6. Summary

**Figure 4:**
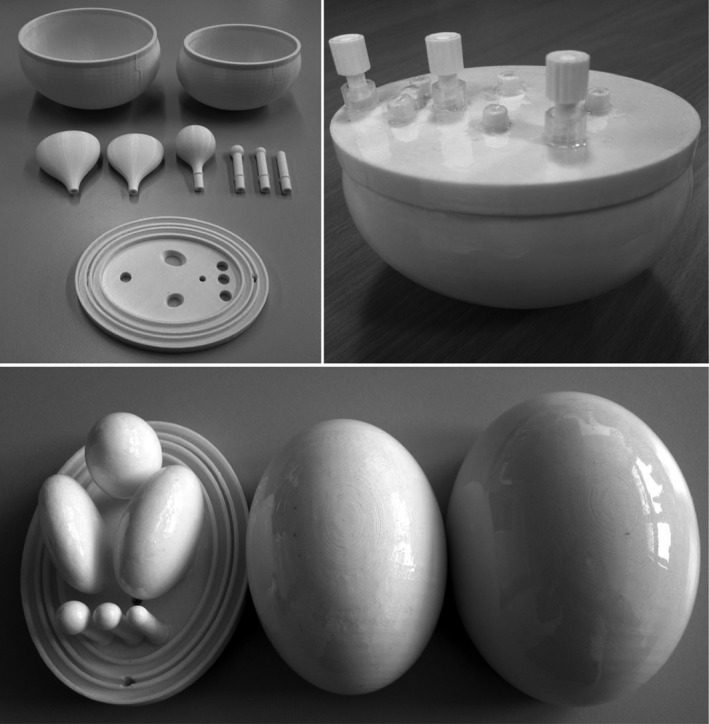
MRI 3D printed phantom.^58^ Figure license: Kasten, et al. #bib2016 #bib3D‐printed Shepp‐Logan phantom as a real‐world benchmark for MRI. Copyright maintained by John Wiley and sons, all rights reserved.

**Figure 5:**
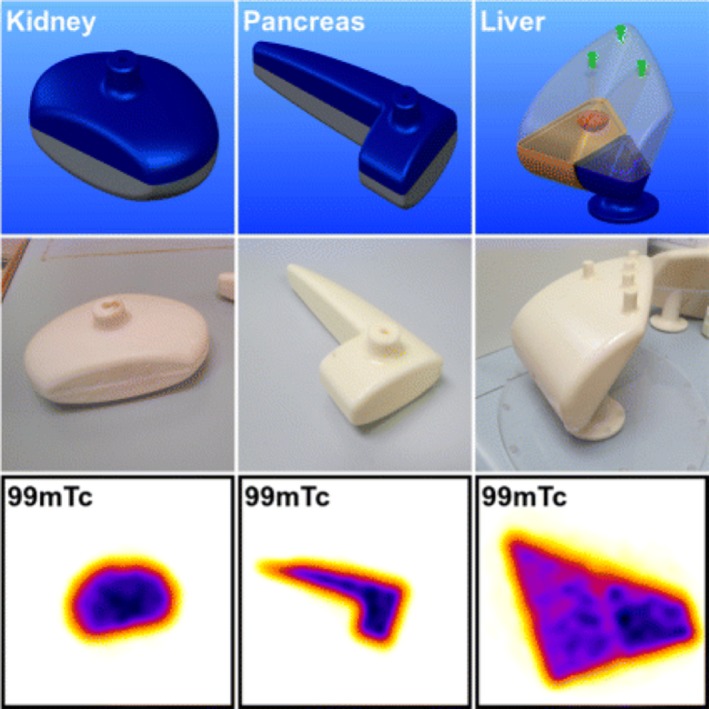
SPECT 3D printed phantom.^34^ Figure license: Robinson, et al. #bib2016, Organ‐specific SPECT activity calibration using 3D printed phantoms for molecular radiotherapy dosimetry. This article is distributed under the terms of the Creative Commons Attribution 4.0 International License ( http://creativecommons.org/licenses/by/4.0/).

**Figure 6:**
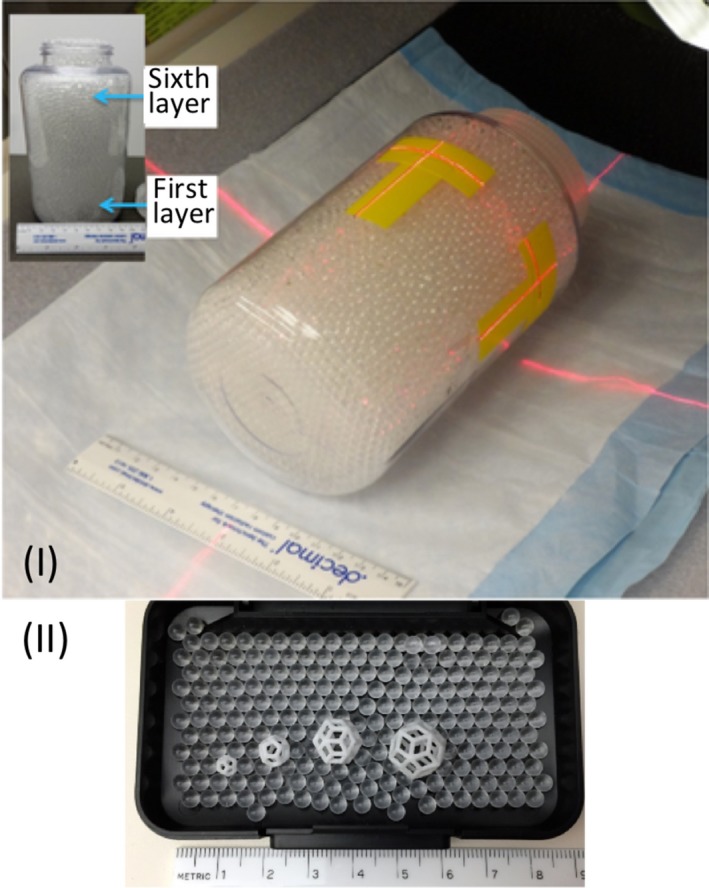
(I) PET phantom, (II) 3D printed lesions.^59^ Figure license: Wollenweber, et al. #bib2016, A phantom design for assessment of detectability in PET imaging. This article is distributed under the terms of the Creative Commons Attribution 4.0 International License ( http://creativecommons.org/licenses/by/4.0/).

**Figure 7:**
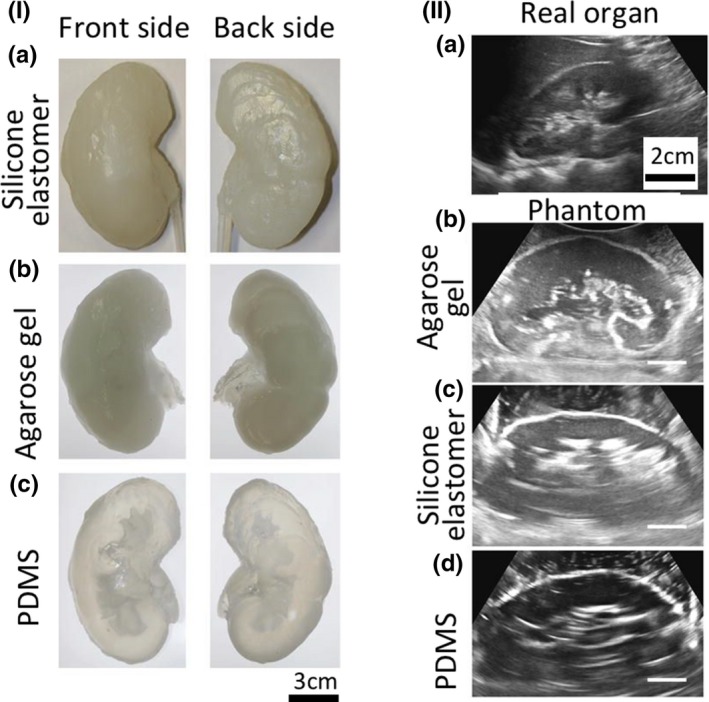
(I) Indirectly 3D printed CT and US phantoms made of (a) silicone elastomer, (b) agarose gel and (c) PDMS, (II) Ultrasound images of (a) real organ, (b) silicone elastomer, (c) agarose gel, and (d) PDMS phantoms.^21^ Figure license: Adams, et al. #bib2016, Soft 3D‐printed phantom of the human kidney with collecting system. This article is distributed under the terms of the Creative Commons Attribution 4.0 International License ( http://creativecommons.org/licenses/by/4.0/)

**Figure 8:**
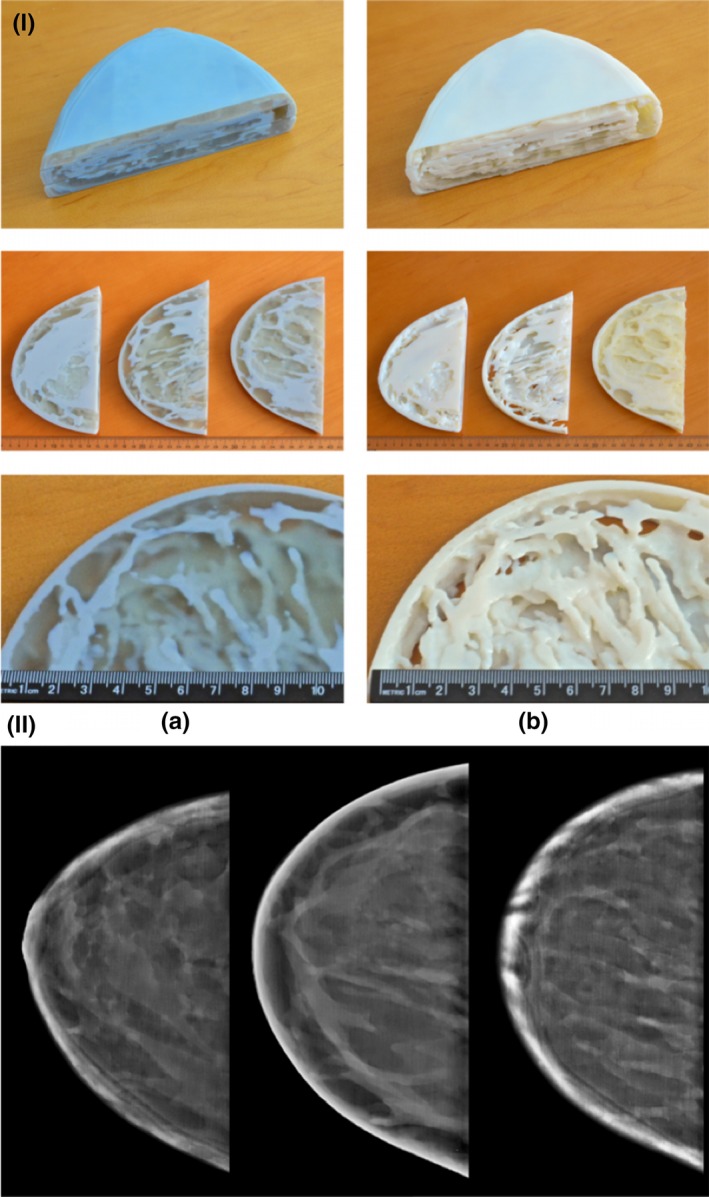
(I) (a) Singlet, (b) Doublet, (II) Mammogram.^48^Figure license: Kiarashi, et al. #bib2015, Development of realistic physical breast phantoms matched to virtual breast phantoms based on human subject data. This article is distributed under the terms of the Creative Commons Attribution 4.0 International License ( http://creativecommons.org/licenses/by/4.0/).

Most articles have compared the phantom images, either with digital images of a patient or another commercial phantom. Table S1 in the Supporting information demonstrates in detail which imaging modality or other device was used by each article to measure the accuracy of the 3D phantom. The majority of the articles used CT scans to assess the accuracy of the 3D printer in producing the phantom. Figures 4, 5, 6, 7, 8 demonstrate different pictures from some of the studies reviewed, which illustrates several 3D‐printed phantoms and their scanned images if available.

### 3.B. Characterization of phantom values

Table 1 summarizes the 3D‐printed materials and the radioactive and nonradioactive solutions used to develop the phantoms. However, almost half of the research articles under review do not mention the properties of those materials. Most of them have calculated and compared the HU of the printed phantoms with a human's organs and tissues. Other properties that some of the articles measured included linear attenuation coefficient, density, acoustic impedance, water absorption, and T_1_ and T_2_ relaxation times.

#### 3.B.1. Torso phantoms

Gear et al.^32^ used common build materials, namely VeroWhite, VeroClear, TangoBlack Plus, to build an anthropomorphic phantom consisting of liver, lungs, and lesions. The HU value of both VeroClear and VeroWhite was almost similar to PMMA (126 ± 15). The HU value of the rubber TangoBlack (96 ± 15) was between the PMMA and water HU values, but closer to the former than the latter.

In addition, Mitsouras et al.^52^ tested 17 materials to identify the most suitable one for 3D printing the vertebrae phantom. The RGD‐525 material was selected, since it was the only one that offered an MRI signal. The average attenuation of this material was 102.4 ± 7.5 HU.

Furthermore, Robinson et al.^34^ used ABS material to 3D print the torso phantom, which includes the spleen, kidney, pancreas, and liver. The mean HU value of this material was measured to be as −54 ± 13 HU, which is close to water's HU value. This material can be used to develop phantoms for MRI scanners, since for MRI, hydrogen nuclei are used because of their large quantity in fat and water.

Another study also printed organs that are included in the torso. Kadoya et al.^22^ developed uterus and bladder phantoms with −35 HU and 90 HU, respectively. However, the phantoms had different HU compared to those of the patient, which were 70 HU for the uterus and 50 HU for the bladder.

Tran‐Gia et al.^57^ developed a kidney phantom using a PLA material to print a cube. The PLA cube had 142 HU, whereas in literature^19^ the human kidney is typically between 30 and 50 HU.

A spinal 3D‐printed model was developed by Javan et al.^38^ who compared the CT number of the phantom with the values of actual tissues. Table 1 of Javan's article demonstrates the minimum, maximum, and average CT number of the cortex bone, spinal cord, nerve roots and muscle. Only the average percentage errors were calculated, which were 15% for cortex bone #bib314% for spinal cord, 383% for nerve roots, and 42% for muscle.

Similar to Javan et al.^38^ Craft et al.^49^ also compared the HU values of the phantom with the image of a real patient at different locations. The HU value differences between them were: 108 (heart) #bib143 (breast), 138 (arm), −128 (left lung), −132 (right lung), and 95 (spine). Note that positive values correspond to the phantom having a greater HU number.

Leng et al.^63^ developed two phantoms representing the liver and brain. The HU of 14 different printing materials were calculated between the range of 70 kV and 150 kV, and the most appropriate materials were chosen. For the liver lesions the rubber‐like material, TangoBlack+ (100 kV acquisition), was selected since it was within the range of CT numbers for contrast‐enhanced liver scans as observed clinically.

Niebuhr et al.^42^ developed a phantom consisting of the pelvic bones, soft tissues (prostate and muscles), adipose tissue, bladder, and rectum. To result in various HU values, different materials were used to represent each part. For example, to represent the outer bone, VeroClear and gypsum were used as the latter contains Ca amounts similar to human bones.

Shen et al.^27^ developed a phantom composed of five organ groups, and for each of these groups the CT numbers were measured: −256 (skeleton), −600 (spinal nerve) #bib350 and 1050 (colon for two groups), 710 and 590 (kidney‐bladder for two groups), as well as 85 for other tissues.

Hazelaar et al.^55^ compared the HU values of the phantom with the corresponding ones of the patient. Si was used to represent the soft tissues; however, the phantom's HU value was much larger than the patient's value: 178 HU vs −43, respectively. Similarly, the gypsum's HU value was much larger than the patient's, since it was 731 HU (compared with 371 HU for the patient). On the other hand, the lung and tumor phantoms had lower HU values than the patient's. Although some of the patient and the phantom's properties were not similar, their images were similar visually.

Joemai et al.^64^ used a different method to assess image quality, called the structural similarity (SSIM) index. This approach is used in the video industry to measure image quality. Even though it is a method not often used in radiology, it seems to be reliable. Positive results have been demonstrated when comparing the phantom image with the patient image. However, it can be used only when there is a digital image available which can be used as a reference.

#### 3.B.2. Head phantoms

##### Computed tomography

Gallas et al.^20^ measured the HU of the materials used to build the head phantom for various voltages, and the measurements demonstrated that as the voltage is increased, the HU is decreased. The materials were water, agarose gel, dosimetric gel, bone liquid, and the 3D‐printed phantom. The HU value of the printed soft bone material varied between 243 ± 16 and 357 ± 24, which changes depending on the tube voltage used.

Negus et al.^41^ developed a brain phantom using PLA material. To build a phantom with similar HU to a brain, several cubes with different fill density were printed and their HU was measured. The HU values ranged from around −400 HU (fill density 50%) to 200 HU (fill density 100%). Since the brain's HU value is between 20 and 40 HU, a fill density of 85% was selected to give 30 HU.

Leng et al.^63^ developed a brain phantom as well as a liver phantom. For the brain phantom (120 kV acquisition), gray matter was printed with PolyJet material RGD8505, white matter was printed with PolyJet material RGD8530, and cerebrospinal fluid was printed with rubber‐like material FLX9895. Although the HU differences between gray matter, white matter and cerebrospinal fluid were similar to the brain CT image of the patient, the actual numbers were different from clinically observed values of brain tissues.

To measure the CT number of the head phantom, Kamomae et al.^51^ measured HU in 20 different locations of the phantom. CT number was compared in both coronal and sagittal directions. The largest differences in CT number regarding the border and the internal regions were ±250 HU and ±100 HU, respectively.

##### Magnetic imaging resonance

In contrast to the other studies, Wood et al.^36^ developed different phantoms with MRI properties, and compared the reflection coefficient of the phantoms with the *in vivo* volunteer at 297.2 MHz. One homogeneous, one heterogeneous, and one spherical phantom were developed and had −18.96, −23.81, −24.87 dB, respectively, while the volunteer produced −23.33 dB.

Saotome et al.^56^ used T_2_ to compare the brain phantom with the brain of a patient. A correlation of *R* ^2^ = 0.955 was calculated, which means that they were almost identical.

#### 3.B.3. Vessel phantoms

Toepker et al.^28^ developed coronary arteries phantoms with stenosis and used FullCure 720 and FullCure 920 materials to print the vessels and the stenotic lesions. These HU numbers are similar to the values that represent fibrous tissue and lipid plaques.

Lai et al.^68^ developed two vessel phantoms: a 3D‐printed one and an agar‐based material. The acoustic properties of these phantoms were compared. The values for the speed of sound of both materials were close, but the attenuation coefficient had large difference between them. For example, the agar‐based phantom had 0.0179 attenuation coefficient, and the printed material had 1.58.

O'Dell et al.^31^ used ABS to develop the vessel phantom. In comparison with the patient's CT image, ABS had 900 HU, while the patient had 1100 HU. Although this was the case, the phantom image exhibits no artifacts produced from motion or variable contrast enhancement, thus, can be a useful gold standard.

#### 3.B.4. Endocrine phantoms

Alssabbagh et al.^40^ used five materials to identify the most suitable to use for the production of the thyroid gland phantom. Among all the potential 3D‐printed materials, PLA was the most appropriate one, since its properties were similar to the thyroid gland. PLA demonstrated 132.4 ± 35.2 HU at 120 kVp, which is close to the associate human number.

#### 3.B.5. Bone phantoms

Rai et al.^66^ 3D‐printed two phantoms—skull and tibia—to represent cortical bone using a photopolymer resin. To replicate any other tissues, other materials like vegetable oil, water, and Gd‐DTPA were used. Resin material demonstrated properties similar to the cortical bone, and it is only visible with ultrashort time echo type of sequences. The T_2_ of resin was 411 μs, which is similar to human bone, but the T_1_ of the phantom was not close to human cortical bone.

#### 3.B.6. Geometrical

##### Computed tomography

Shin et al.^62^ measured the HU of 16 materials of filament phantoms, resulting in greater variations between materials. The 3D‐printed materials were tested at 80 #bib100 #bib120, and 140 kV, resulting in variations of HU between −61.4 (100 kV) and 345 (80 kV).

Madamesila et al.^60^ developed two cylindrical lung phantoms of low and high densities. Prior to the development of the lung phantoms, different infill pattern phantoms were 3D‐printed, such as grid, honeycomb, concentric lines, and triangles. Each pattern resulted in different HU values, and a calculated calibration curve was developed and used for the 3D‐printed lung phantom development.

Ceh et al.^29^ compared the CT numbers of the patients and the phantom for different anatomical features. The HUs of the phantoms were approximately 2.61 #bib2.56, 2.82, 2.53, and 2.63, and the corresponding HUs of the patient were 1.04, 1.54, 1.09, 1.04, 1.75, which represented the zygomatic bone, the middle turbinate bone, the zygomatic bone (lateral), the upper mandible, and lower maxilla, respectively. There is tiny difference between them, with an average percentage error change of 1.13%.

Furthermore, Dancewicz et al.^61^ tested the CT numbers of the phantoms against a commercial phantom, called Gammex, at 80 and 120 kVp, for CT image acquisition. They demonstrated substantial variations between −943 ± 14 and 3568 ± 532 HU at 80 kVp, as well as variations between −916 ± 1 and 7257 ± 24 HU at 120 kVp. Some of the printed materials demonstrate similar HU values to the commercial phantom. For example, the Gammex insert Lung‐300 had −746 ± 19 HU and the 30% ABS‐based phantom had −760 ± 13 HU at 80 kVp. In addition, the commercial and 3D‐printed phantom imaged with megavoltage CT and the printed phantom demonstrated variations of HU value between −842 ± 1 and 739 ± 6.

The phantom developed by Seoung et al.^30^ was compared with the American Association of Physicists in Medicine (AAPM) CT phantom, and against AAPM CT evaluation criterion values. Several parameters were measured, such as noise (below 7 HU), water attenuation coefficient (0±7 HU), image uniformity (±5 HU), spatial resolution (1.0 mm), and contrast resolution (6.4 mm).

Lin et al.^39^ 3D‐printed a trabecular bone and cubic phantoms. The CT number of these phantoms was measured using different slice thicknesses. The HU value varied depending on slice thickness, between the range of 0 and 120 HU. The cubic phantom at 0.5 mm slice thickness was 112.3 ± 3.5 HU.

Wollenweber et al.^59^ placed several 3D‐printed features in a bottle and scanned the bottle using a CT scanner. The HU values were in the range of −160 and 240 HU.

As already mentioned, Hamedani et al.^54^ printed several phantoms to address specific issues using each of them. Cylinders with different variations of BaSO_4_ were 3D printed to check how the HU value changes according to the BaSO_4_ weight. It is demonstrated that using greater weight of BaSO_4_, the HU number also increases. The HU range offered by changing BaSO_4_ concentrations was between −31 and 1454 HU. In addition, cortical and cancellous bones were printed by changing parameters of both the infill percentage and the shell thickness. Considering the cortical bone, the HU value of 1 and 2 mm, largely differ from the desired value. On the other hand, cancellous bone HU values were much closer to the target values. For instance, regarding the 20% infill, the error between the measured and the target value was −8.7%, as the phantom's HU value was 200 HU, and the patient's was 184 HU. The 10% infill had a larger degree of error, as the phantom had 131 HU, and the patient image had 92 HU.

##### Ultrasound imaging

Fuzesi et al.^47^ tested ABS, PLA, and photopolymer to see if they can be used as scatterers in ultrasound imaging. Acoustic parameters such as acoustic impedance, attenuation, and speed of sound were investigated at 2.25 #bib5, and 10 MHz. Although, some of the results are close to literature values, and some not, these materials demonstrated positive results to be used as scatterers.

##### Multimodal imaging—CT, US, MRI

In et al.^69^ developed cylindrical 3D‐printed silicone multimodal imaging phantoms with variable water content and hydrophilic silicone content to mimic the human liver. The phantoms were tested in CT, MRI, and US imaging modalities. Regarding the US properties (speed of sound and attenuation coefficient), the phantom showed different values in comparison to the human liver values reported in literature.^69^ The speed of sound in the phantoms was between 906 m/s and 1275 ± 40 m/s, where in the human liver it is 575 ± 11 m/s. In addition, the attenuation coefficient of the phantom was lower than that of the human liver, but they are measured at different frequencies. The MRI T_1_ and T_2_ were used to compare the phantom image with the human liver image. Human liver has T_1_ value equal to 812 ± 64 ms and T_2_ equal to 42 ± 3 ms, and the phantom had T_1_ equal to 448 ms and T_2_ 40 ms. The CT number of the phantoms was measured at 120 kVp. Both phantoms had 10% hydrophilic silicone, but one contained no water and the other one comprised 20% water. The phantom with no water had 142 HU, and the one with water had 77 HU. It is worth highlighting that when there is a higher content of hydrophilic silicone in the phantom, the CT number tends to become higher.

Based on all the findings, it appears that several studies have incorporate some additional materials that are not 3D printed to reach a realistic variation in HU values which are observed in humans.

### 3.C. Fluids and radiotracers

**Figure 9:**
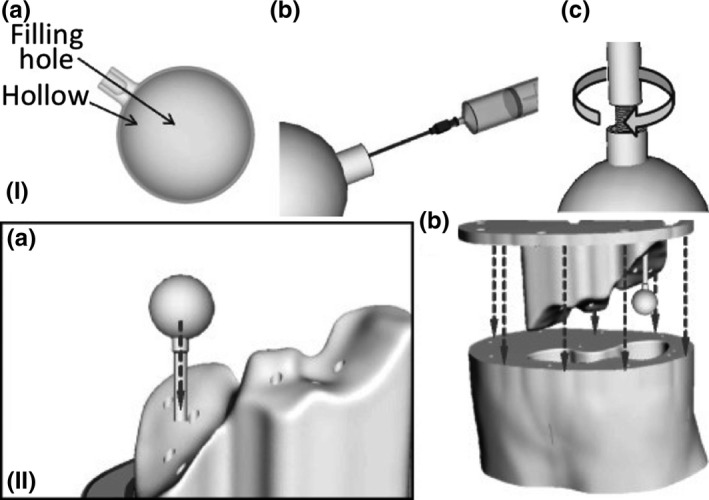
(I) (a) Lesion design, (b) Lesion filling, (c) Connection port, (II) (a) Lesion and support rods placement at the phantom base, (b) Phantom base fitted to the phantom body.^32^ Figure license: Gear, et al. #bib2016, Abdo‐Man: 3D printed anthropomorphic phantom for validating quantitative SIRT. This article is distributed under the terms of the Creative Commons Attribution 4.0 International License ( http://creativecommons.org/licenses/by/4.0/), Changes: Addition of (I) and (II) on top of the pictures, in their original form these two figures are separated.

**Figure 10:**
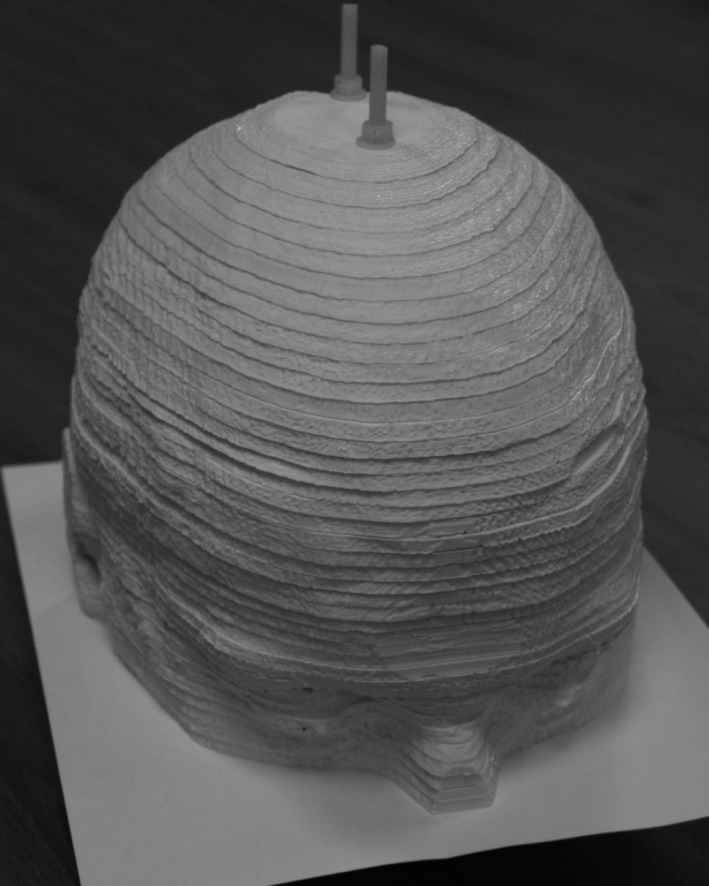
Sub‐resolution sandwich phantom with radioactive paper sheets between each slab.^41^ Figure license: Negus, et al. #bib2016, Technical Note: Development of a 3D printed subresolution sandwich phantom for validation of brain SPECT analysis (Copyright by John Wiley and sons).

**Figure 11:**
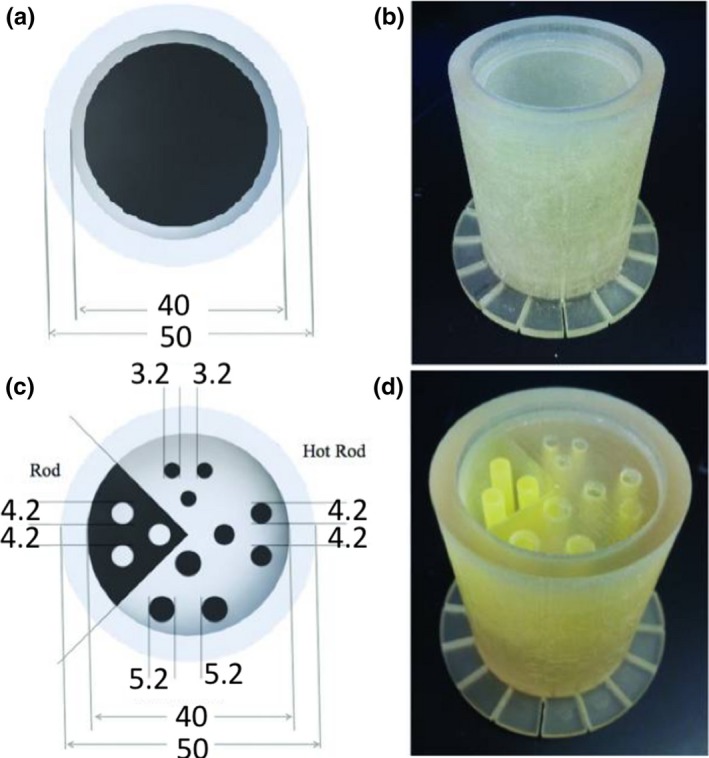
(a) Diagram and (b) photograph of 3D printed PET/MRI normalization phantom. (c) Diagram and (d) photograph of a 3D printed PET/MRI resolution phantom with hot and cold rods.^10^ Figure license: Bieniosek, et al. #bib2015, Technical Note: Characterization of custom 3D printed multimodality imaging phantoms. (Copyright by John Wiley and sons).

**Figure 12:**
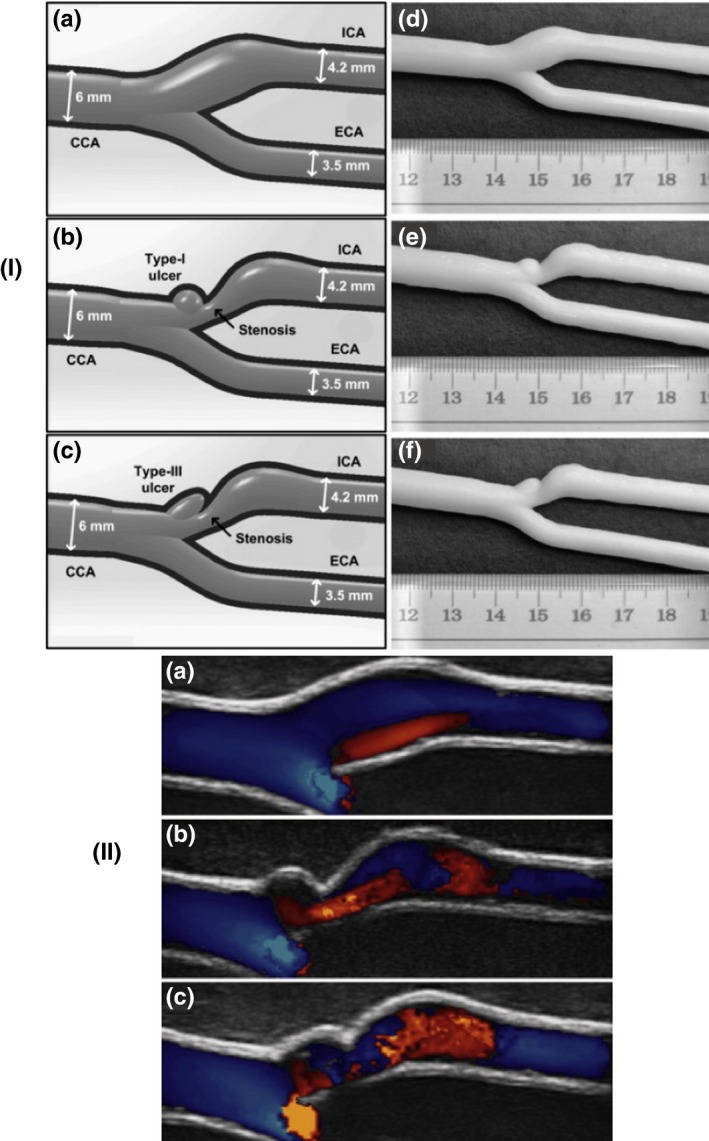
(I) Three different geometries of carotid bifurcation vessel tubes, (II) Ultrasound flow images for the different geometric phantoms.^68^ Reprinted from Ultrasound in Medicine and Biology, Vol. 3, Lai SSM, Yiu BYS, Poon AKK, Yu ACH, Design of anthropomorphic flow phantoms based on rapid prototyping of compliant vessel geometries #bib1654‐1664, 2013, with permission from Elsevier.

Table 1 demonstrates which papers have used radiotracers inside their phantoms for either SPECT or PET imaging. Several different approaches have been used to fill the phantoms with a radiotracer. Gear et al.^32^ developed spherical inserts of different diameters, injected the radioisotope inside those inserts and attached them using a detachable support rod to the base of the phantom, referred to Fig. 9(II). In this phantom, there is a hole at the connection point of each sphere, which allows the insert to be either filled or emptied easily using a gauge needle, as demonstrated in Fig. 9(I). VeroClear FullCure 810 material was used to 3D‐print the inserts. This material is transparent and helps visualize any solution that is poured into the phantom.^18^ Robinson et al.^34^ and Woliner van der Weg et al.^72^ used an alternative approach as they designed each compartment of the phantom separately. Some of the compartments were designed with one opening, as demonstrated in Fig. 5, aiming to be filled with a solution, such as radiotracer or “bone” material K_2_HPO_4_.^17^, ^18^, ^34^, ^72^ A completely different approach was used by Negus et al.^41^ who operated an FDM 3D printer to build transaxial slabs, and operated a standard Hewlett‐Packard Officejet Pro 8100 printer to print radioactive paper sheets.^41^ To do that, the radioisotope was inserted in the ink cartridge of the printer. The slabs and the radioactive sheets were assembled to develop a complete sub‐resolution sandwich phantom, as shown in Fig. 10. Wollenweber et al.^59^ used a bottle as the phantom's case and placed solid acrylic spheres and the 3D‐printed polyhedral features. Then, the radiotracer was poured in the bottle, filling the empty spaces that left from both spheres and features. This can be seen in Fig. 6. A team at Stanford University 3D printed cylinders (Fig. 11) with hollow wells, which were filled with imaging agents and radioisotopes.^10^, ^76^ Kasten et al.^58^ Gallas et al.^20^ and Niebuhr et al.^42^ also created an opening on the phantom's compartments and filled it with a solution. Alqahatni et al.^73^ poured water solution mixed with ^99m^Tc inside the printed thyroid gland, which had two syringe filling valves on top. Also, Tran‐Gia et al.^57^ used a syringe to fill the radioactive solutions into the 3D‐printed kidney phantom via a small 3D‐printed tube. Various radioactive solutions were used (^99m^Tc, ^177^Lu, ^131^I) to fill in a range of kidney and sphere phantoms: those of a newborn, a 1‐year old, a 5‐year old and an adult. Figure 4 demonstrates a brain phantom composed of compartments that are loaded with either radioactive and/or nonradioactive solutions. For example, agarose gel, water, K_2_HPO_4_, agar‐based mixture, olive oil, and vaseline are nonradioactive solutions, which represent brain gel, ventricle liquid, bone liquid, prostate tissue and adipose tissue, respectively.^20^, ^42^ Other studies, such as Lai et al.^68^ and Morais et al.^46^ used nonradioactive solutions to mimic the blood flow, which is an important physiological parameter. For the generation of the fluid flow, a gear system was used to control the flow in each phantom. These phantoms have a significant role, since blood is a fundamental element of the human body that affects all body functions.^77^ Figure 12 demonstrates an example of a flow phantom developed by Lai et al.^68^ and the corresponding US image.

## 4 Discussion

This review focuses on the production of physical phantoms that are used in medical imaging to simulate human or animal tissue in experimental procedures.^1^ One of the new manufacturing procedures of developing phantoms is 3D printing technology, which offers potentially greater realism and pluralism.^7^ Therefore, this paper reviews 3D printers that have been used in terms of their resolution and printed material properties. Considering the material properties, the review is focused on whether the materials used are able to mimic the acoustic and other properties of the tissues and organs, mostly by assessing HU, as well identifying the feasibility of including solutions inside the 3D‐printed materials.

### 4.A. Characterization of 3D‐printed phantom spatial accuracy

**Figure 13:**
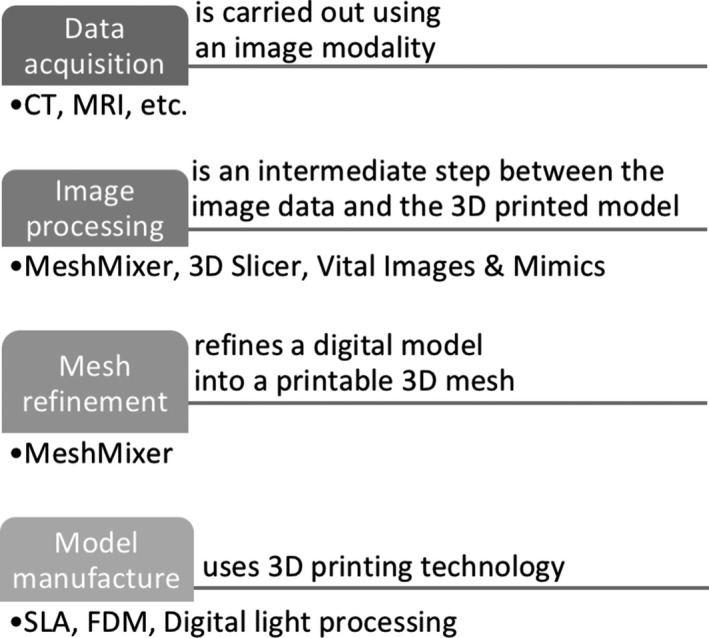
Procedures that affect the accuracy of the phantom.

The reviewed papers illustrate the development of anthropomorphic, animal, or geometrical phantoms. Anthropomorphic and animal phantoms have more complex shapes when compared to standard geometrical phantoms; however, all can be used to identify whether the resolution of the printer is sufficient. To achieve the best possible image outcome from the phantoms, the details of their features have a significant role associated with the resolution of the 3D printer. From Section Results, it is demonstrated that most of the 3D printers have printed phantoms with almost the same dimensions as the original ones. The identified differences are due to the following reasons: (a) data acquisition, (b) image processing, (c) mesh refinement, and (d) model manufacture.^78^ These procedures are used to transform a medical image data set to a 3D‐printed phantom. In each step, there is the potential to create geometric distortions and errors in the printed model as shown in Fig. 13.^78^

**Figure 14:**
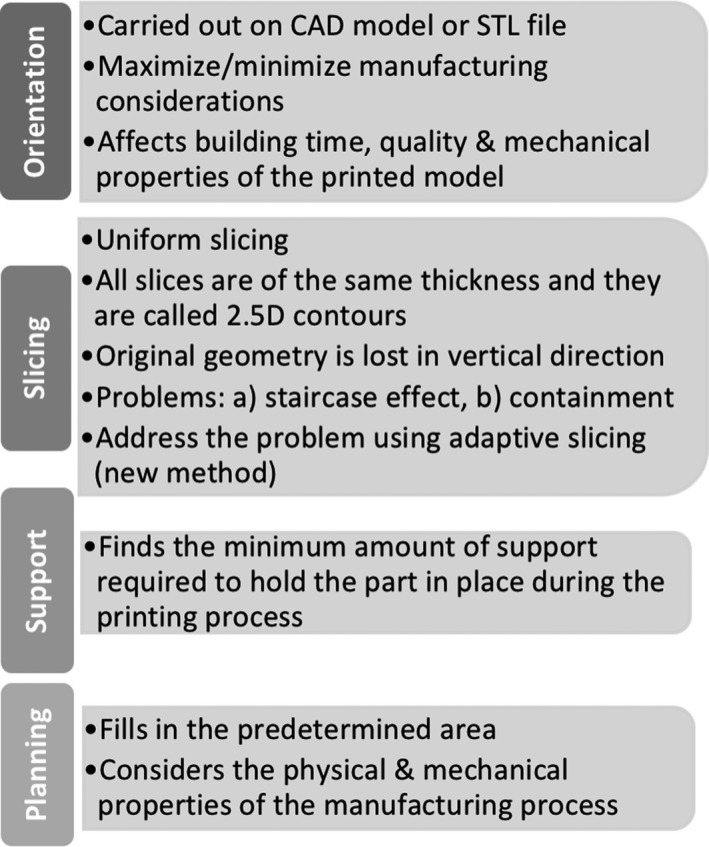
Diagram of the steps involved in the 3D printing process.

Data acquisition is most commonly carried out using a CT scanner because of its good spatial resolution, high contrast, and signal‐to‐noise ratio, which improve the differentiation of the structures and decrease partial volume effects.^9^ Furthermore, image segmentation is used to partition the captured image into numerous labeled regions to locate boundaries and objects in images to segment the regions of interest and output an STL file.^33^ To do that, there are several image segmentation techniques; however, no single segmentation technique exists to be suitable for all medical images. The next step is mesh refinement, which is used to repair any errors created during the segmentation step, prior to printing and to smooth the mesh model's surface since “staircasing” errors might occur from the resolution of the original image, and lastly to append the model by either removing unnecessary parts or adding other useful structures.^33^ For example, Gear et al.^32^ made the mesh smoother by removing the image pixelation, which in turn altered the phantom prior to printing. Furthermore, there are several 3D printing technologies available to manufacture the 3D‐printed phantoms and each technology has its own benefits and limitations that affect the 3D‐printed end product. Some of the limitations are further described, for instance, there is a possibility of leftover support material, which means that if a solution is filled into the phantom it might take up less space than intended. If this is measured, the volume measurement will be lower than the original volume.^18^ In addition, printers have physical constraints which may affect the phantom and lead to final differences from the prototype.^34^ A few articles have undertaken only qualitative comparison, which does not create reliable conclusions regarding the printer's actual resolution. Quantitative comparison is more representative since the percentage difference of any desired property can be calculated. The phantom is compared against the original MRI and/or CT patient scan, or another physical, computational phantom, or even with the original dimensions of the geometrical shape that was developed using CAD software. Although quantitative results are directly measurable, their values in these cases often originate from medical images that have been created digitally or physically with the use of digital or physical calipers. Geometrical parameters, for example, thickness, volume, and length, are numerical measures used to represent the accuracy of 3D‐printed phantoms. However, these measures may represent several millions of voxels, therefore, if there is a small error in each voxel, then the errors will accumulate and the whole 3D‐printed phantom will have different dimensions. For instance, Craft et al.^49^ developed a phantom which consisted of 11 slices and observed such discrepancies. Furthermore, Mitsouras et al.^52^ scanned the printed phantom with CT and MRI and then tested its accuracy using both CT and MRI modalities. They identified that the dimensions in the two modalities were different, with the MRI demonstrating much larger differences in the phantom's dimensions compared with the CT. This is an example which shows that imaging modalities have their own limitations as well, and that phantoms might also depend on the technique being used to perform the original scan.

In general #bib3D‐printed phantoms do not have the complexities that the real human body has, since they need to be modified to be appropriate for the properties of the printer. Some of the printers’ vertical resolutions reach 16 μm, or around 1600 dpi, which offers sufficient results. Although thinner layers offer the finest details, they require more repetitions and thus it takes longer to print the model. The Z resolution (μm) of the printer is inversely proportional to printing time. Moreover, thinner layers may result in more errors and artifacts. The final result does not depend solely on the Z resolution, but also on the XY resolution and on the model. After all, the aforementioned 3D nominal resolution values are claimed by the manufacturers; however, they might not necessarily reflect reality. In addition to resolution, accuracy plays an important role for the finished model. Accuracy highly depends on the imaging technology used to scan the patient, and later the printed phantom, the software used to design or process the phantom, the materials used to build the model, and the 3D printer used to print the phantom. Dimensional accuracy, shrinkage, and support requirements are variables that are used to quantitate accuracy. For example, the accuracies claimed by the manufacturers are relative to well‐designed parts on a well calibrated machine. In addition, support material affects the accuracy of the surface finish, since it often has to be removed.^1^^,^ 2

### 4.B. Characterization of phantom imaging values

Images are used to visualize the human body *in vivo*. Several measurements, such as the linear attenuation coefficient, HU, acoustic impedance and water absorption are useful to demonstrate numerically what is seen in a voxel based on the images captured. Ideally, a single phantom should be identical to its human counterpart across all these parameters, but this is not yet possible. Until now, most phantoms might only represent a handful of measured parameters. Most materials used by the researchers of the reviewed papers demonstrated HU close to water, with the exception of Gallas et al.^20^ who achieved much greater variation in HU since several human brain surrogates were developed. This suggests that by altering the chemistry of the 3D‐printed materials, for example, by adding other solutions like pigmented binders of different concentrations, greater variation in properties can be achieved. In addition, if a greater range of material properties is tested, rather than just the traditional ones, it would be useful to determine other radiological properties. Most of the investigations have made use of commercially available materials for their phantom, which means that they can be easily reproduced by other groups.

HU is one of the most common parameters measured. Bibb et al.^79^ measured the HU of 14 commercially available materials from several additive manufacturing machines. In addition, Yoo et al.^25^ measured the HU of 12 materials, most of which resulted in negative values although one had a positive value close to compact bone. The reason the HU changed is because the binder was altered by the inclusion of NaI, and because different binder colors were used. On the other hand, Gallas et al.^20^ and Niebuhr et al.^42^ 3D printed the frame of the phantom, but used different surrogates to achieve the same properties as human tissue. For example, they both used K_2_HPO_4_ as a bone liquid, and agarose gel as brain gel and prostate tissue. Furthermore, Nikitichev et al.^67^ Wood et al.^36^ and Saotome et al.^56^ used agarose gel to represent soft tissue. These results suggest that 3D printing technology needs further refinement regarding the radiological properties of the materials. The majority of printer manufacturers measure mechanical properties such as tensile strength, elongation at break, modulus of elasticity, hardness, flexural strength, and others, but to the best of our knowledge manufacturers have not made available the properties of the materials that are beneficial to different imaging modalities. These would include speed of sound, acoustic impedance and attenuation coefficient, which are important parameters for the US scanner. Pacioni et al.^80^ and Jacquet et al.^81^ measured all three parameters, however, the results should be further improved to achieve values closer to human tissues. It is important to mention that Nikitichev et al.^67^ Adusumilli et al.^45^ and Morais et al.^46^—who all use a US imaging modality—did not measure any of those parameters, except Adusumilli et al.^45^ who measured only the speed of sound. Furthermore, in MRI scanning the T_1_‐ and T_2_‐weighted images are the most common sequences used, and thus some of the papers have measured these two parameters. T_1_‐ and T_2_‐images are produced using short and long TR and TE times, respectively, where the brightness and contrast are determined via the T_1_ and T_2_ properties of tissue, respectively. For example, increased levels of water in tumors appear dark on a T_1_‐image and bright on a T_2_‐image. Niebuhr et al.^42^ compared both T_1_ and T_2_ to human values from literature, and the T_2_ values of the phantom were closer to real values in comparison to T_1_, however, both properties need further improvement. In addition, this phantom was used to represent the pelvic bone, soft tissues, organs, muscles, and adipose tissue. All phantom parts appeared slightly different in the MR images when using T_1_ and T_2_. Similarly, Gallas et al.^20^ demonstrated differences in images captured at T_1_ and T_2_, which they appeared to be major for the cerebrospinal fluid surrogate and polymerization gel dosimeter. Wood et al.^36^ also compared the scattering parameters, such as the reflection coefficient, of different types of phantoms acquired with a 7 T scanner and compared them to the original volunteer images which were acquired with a 3 T scanner.

Currently, the materials used to develop 3D‐printed phantoms have only a few of the essential properties to develop a realistic multimodal/multiparametric phantom. Even though some materials represent accurately different tissue properties, they only do so for a specific imaging modality. It has been difficult to identify materials suitable for all imaging modalities. However, this new field has great potential to achieve more versatile phantoms.^10^, ^16^, ^17^, ^18^, ^19^, ^20^, ^21^, ^24^, ^32^, ^42^, ^52^, ^59^, ^69^, ^72^, ^75^, ^76^, ^80^

### 4.C. Fluids and radiotracers

Different solutions, such as radiotracers, water, and agarose gel have been poured into the 3D‐printed phantoms manually. None of the authors placed any solution inside the 3D printer beforehand, except Negus et al.^41^ who mixed the radiotracer solution with the ink of an standard inkjet printer. The approaches described above used more efficient methods than in standard phantoms, where the whole phantom had to be taken apart, the solutions re‐poured and the phantoms reassembled. Although the new approaches are more efficient and faster, they faced common issues, such as air bubble formation due to the quick pouring of the solution into the phantom. Wollenweber et al.^59^ and Kasten et al.^58^ mentioned that they detected air in the produced images. This issue could be resolved if the 3D printer could directly print radioactive material. Another issue is that different radiotracers act differently, therefore, it would be difficult to “3D print” materials with radiotracer. 3D printers may take long period of time to print a complete phantom (for example #bib154 h)^32^ therefore, it is not sensible to “print” a radiotracer which has much shorter half‐life, for example 110 min for ^18^F.^82^ Printing with longer lived radiotracers is more appropriate, but the printer would need special care as it will have radioactive parts which could complicate procedures such as maintenance and storage. Until today, it has been challenging to include a solution such as a radiotracer or a nonradiotracer inside the 3D printer to be used for printing. To achieve this, changes focused on the medical imaging field in 3D printing technology are essential, as discussed later on. In addition, future investigators could possibly test whether solutions, either radioactive or not, caused any alterations to the physical phantom properties. None of the reviewed papers mentioned any issues associated with these solutions.

### 4.D. Limitations

3D printing technology has revolutionized manufacturing and offers a great potential for the development of phantoms for the variety of imaging modalities. Nonetheless, several challenges have been identified from the specific articles reviewed (4.D.1) and from other articles (4.D.2).

#### 4.D.1. 3D‐printed phantom limitations as identified in the reviewed articles

Most 3D printers’ manufacturers are testing the mechanical properties of the materials such as Young's modulus, hardness, and other mechanical properties, but do not consider the radiological or acoustic properties of the materials. This does not help boost the use of 3D printing in the medical imaging field, as it becomes time‐consuming to test all available additive manufacturing materials to identify the desired properties for each phantom. Moreover, most of the authors have examined only a small number of 3D‐printed materials. This is a considerable limitation since materials that may have better material properties have not yet been examined. In addition, the materials that have been tested by different researchers are limited to specific properties. For instance, Niebuhr et al.^42^ used several materials to represent a pelvic phantom. Two of these materials, gypsum and VeroClear, showed good results when tested with a CT scan, but they showed poor results when tested with an MRI scan. This suggests that currently it is not straightforward to use only one phantom for multimodality imaging. Another noteworthy limitation is the geometry and the type of materials of the phantoms, which seems not to be adequately representative of human tissue. For example, in several instances phantoms have either cylindrical or rectangular shapes, or a single material is used to represent different tissue layers, or the radionuclide solution is uniformly distributed, which is not the case in reality. In addition, authors who developed small geometrical shapes or smaller anthropomorphic phantoms than the organs of humans may experience variations in appearance if printed in actual scales.

A few papers identified include flow motion,^68^, ^83^, ^84^ however, the respiratory and cardiac motions of the patient are not included. Therefore, these phantoms are not completely realistic to test motion correction algorithms. All types of motion have a significant and unique role in the human body. If they are considered in the development of imaging phantoms, the measurements taken would be much more realistic than the existing results. Flow phantoms are of great importance since they represent blood flow, which is fundamental for the health of humans. These phantoms could be used to measure arterial blood flow, the volume rates of blood flow in several organs and many other measures.^77^

In addition, only a few papers discuss the durability of the phantoms to withstand scanning protocols, multiple assembly and radioactive solutions, as well as about their cleaning process. This is important information since there is a need to use the 3D‐printed phantoms more than once in different modalities, and sometimes with radioactive solutions.

Furthermore, some researchers could not eliminate the air bubbles produced from the liquid mixture used for mimicking the different human tissues, which creates artifacts in the produced images. Also, the removal of the support material sometimes damaged the phantoms, which is not desirable as the liquid mixtures that are inside the phantom may leak. Jacquet et al.^81^ used the support material to mimic a specific human tissue, therefore there was no need to remove it, which could have avoided possible damage to the phantom. Lastly, most of the papers did not include the costs of the materials, the amount of time needed to print the phantoms, the printing parameters and conditions of the 3D printers, thus most of these details, such as costing and time, are not mentioned throughout this report.

#### 4.D.2. 3D‐printed phantom limitations as identified in the extended literature

Shape optimization, printing methodologies, pre‐ and post‐processing, are some of the limitations that are often ignored by manufacturers.^85^ In this subsection, two important limitations that affect phantom development are discussed.

CAD is the current software used for 3D printing, even though it was not designed for this purpose. The models created by CAD software are a combination of constructive solid geometry and boundary representation. These techniques have been successfully used for other manufacturing objectives, however, they currently limit what can be done using 3D printers, for example phantom geometry. In addition, complex or large designs cannot be produced as a single model, since they require special design considerations. Therefore, this might result in even more complex products.

For the development of the final product, the pre‐ and post‐processing steps are critical. Prior to the printing process, the final CAD model is approximated by an STL file format with sets of planar triangles to be read by the 3D printers. Due to this conversion, the accuracy of the final model is often compromised, geometrical detail is missing, and misaligned facets and redundant triangles may be generated. In Fig. 14, we describe the four tasks that would be helpful in optimizing the 3D printing process.

After the model is printed, it might suffer from the staircasing effect. To overcome this problem, melting, traditional machining, and acetone finishing are methods used to improve the surface quality. The final product might get disrupted if the support material is not removed carefully.

“Ten challenges in 3D printing” by Oropallo et al.^85^ is recommended as a useful paper to refer to regarding a detailed overview of the current 3D printing technology challenges. Furthermore, there are useful methodologies in the report of the Food and Drug Administration (United States of America) guidelines of technical considerations for additive‐manufactured medical devices.^3^ Some of these considerations may be helpful in creating a consensus for the development of high quality standards for 3D printing of imaging phantoms. For example, the FDA suggests specific steps which if followed, may result in more reliably printed models. Perhaps there is a role for scientific associations such as the American Association of Physicists in Medicine, the European Federation of Organisations for Medical Physics and the Institute of Electrical and Electronics Engineers to organize and coordinate appropriate task groups which will aim to compile guidelines, and standardize the procedures for developing 3D phantoms, as well as convince manufacturers to make key information about the materials used for the development of realistic phantoms openly available.

### 4.E. Future directions

In terms of future work, there are two main aspects that need to be considered to develop phantoms that will be used in several imaging scanners. The first aspect is to identify or build materials that have several properties that serve each scanner. This will enable the development of a single phantom that will have the potential to be used in CT, mammography, MRI, PET, SPECT, and US. Due to that, the possibility of using these scanners simultaneously will provide great advancements in medical imaging. The second direction is the development of intrinsically moving phantoms. Humans and animals exhibit cardiac and respiratory motion, which create artifacts in the produced images. Therefore, there is a need to incorporate motion in the phantoms for testing motion correction algorithms. The following two sub‐sections demonstrate two future routes to produce phantoms with higher levels of realism.

#### 4.E.1. Toward moving soft 3D‐printed phantoms

Soft and flexible materials are the key solutions to develop movable phantoms. Soultanidis et al.^86^ constructed a cylindrical phantom made of cryogel, which contains another smaller “cold” cylinder that is made of PVA, Gd solution, and radioactivity. To make the phantom to move, a stepper motor was used for the production of sinusoidal motion through a piston. It is important to consider that the motion produced can be made more complex, as it can be programmed by the user. Alternatively, a dynamic thorax phantom was constructed by Fieseler et al.^87^ which offers both respiratory and cardiac motion. The plastic thorax phantom consists of inflatable silicone lungs, a liver compartment and a left ventricle model that deforms. A pneumatic piston was used to move the phantom's diaphragm. This phantom offered near‐realistic PET‐MRI images—with both respiratory and cardiac motion. Therefore, it can be used to improve motion correction algorithms, as well as software‐based simulations. These phantoms were not 3D printed, however, current 3D printing technology offers great variations of rigid and soft materials. Therefore, soft movable phantoms can be 3D printed either directly or indirectly. For example, Drotman et al.^88^ at the University of California developed a complex 3D‐printed soft‐legged robot which is able to navigate challenging terrains.

#### 4.E.2. Toward 3D bioprinted tissue phantoms

A completely different way to produce phantoms that offer physiological realism is to 3D bioprint synthetic tissue models. This means that different cells collaborate, achieving functionality which is not generated by single compartments. Booth et al.^89^ developed a 3D printer that creates models of communicating aqueous droplets in arranged patterns. These structures adopted several properties, for instance to conduct or to fold electrical signals. With the current advances in 3D printing technology, the properties of those materials were improved in such a way as to become true synthetic tissues. This was achieved by performing sophisticated functions like protein synthesis. In addition, synthetic tissues were energized and controlled from external sources such as light. Furthermore, Serpooshan et al.^90^ reviews the progression of the design and printing of 3D‐bioprinted cardiac tissues. Technical and biological complexities, for example tissue architecture and vasculature design, cells and biomaterials’ selection, cell function and viability. Bioprinting offers tremendous opportunities that will provide a new era in 3D printing technology and consequently 3D‐printed phantoms for imaging. We envisage that future phantoms will have more biological realism and will be used to study the function, diffusion, physiology and kinetic properties of synthetic organs. A recent review article by Wang et al.^91^ has been published regarding 3D‐printed biostructures for regenerated organ and tissue, as well as medical phantoms. The review discusses 3D‐printed tissue‐mimicking phantoms, radiologically relevant phantoms, and physiological phantoms. In general, it gives a comprehensive overview about several 3D‐printed medical phantoms that are currently being developed.

## 5 Conclusion

3D printing technology is a rapidly emerging field and it is now used for the development of phantoms in medical imaging. It is a cost‐ and time‐effective process that allows for the creation of more complex and detailed phantoms. Therefore, the aim of this review was to examine the different 3D printers that have been used until today to print phantoms for imaging and to identify whether the printers’ resolution and materials represent acceptable human tissues’ and organs’ properties, respectively. The papers that have been reviewed used 3D printers with up to 16 μm vertical resolution, however, even if the resolution of the printer is relatively high or low, the properties of the 3D‐printed materials have a significant role in the development and employment of such phantoms. According to the results obtained from our literature survey, the resolution of the 3D printers used is able to develop detailed phantoms. However, better coverage of materials would have been helpful to develop realistic phantoms, achieving sizes of tissues and organs comparable to those of humans and animals. The materials of the printers are yet to demonstrate the extent of what is required for tissues or organs so that they can be used in multimodality hybrid imaging. In addition, there have been only limited discussions or investigations on how the radioactive solutions may affect the properties of the 3D‐printed materials. Generally, there is a great potential for growth in this area, but companies which develop the printers and the associated materials could consider a wider range of material properties useful in medical imaging. Ideally, a 3D printer dedicated for printing imaging phantoms would be very useful. This will enable researchers to choose which printers and materials are suitable for the development of phantoms. 3D‐printed phantoms will be pivotal in the evolution of the medical imaging field, as they give the opportunity to test and improve several aspects of the scanners’ hardware and software. At the moment, it is feasible to use some specific phantoms for two or three imaging modalities, however, the technology requires further improvement for use with multimodality systems.

## Conflicts of interest

The authors have no conflicts to disclose and they reported all papers that satisfied the specified relevant criteria, but if some papers are missing, this was not intentional and they wish to proactively apologize to any authors.

## Supporting information


**Table S1.** Accuracy measurements of the phantoms and image processing software.

## Notes

1 See web page: https://www.3dhubs.com-knowledge-base/dimensional-accuracy-3d-printed-parts

2 See web page: https://re3d.org/3d-printing-dimensional-accuracy/

3 See web page: https://www.fda.gov/MedicalDevices/NewsEvents/WorkshopsConferences/ucm587582.htm
