# Supplementary material for: Recent advances on the development of phantoms using 3D printing for imaging with CT, MRI, PET, SPECT, and ultrasound
Source: Med Phys. 2018 Jul 24;45(9):e740–60. doi: 10.1002/mp.13058 (PMC6849595; doi:10.1002/mp.13058)
Supplement: Supplementary file 1 — Table S1. Accuracy measurements of the phantoms and image processing software. [file MP-45-e740-s001.doc]

Table 3: Accuracy measurements of the phantoms and image processing software

| ***First Author*** | ***Year*** | ***Accuracy measurement (Imaging modality / Other device)*** | ***Image processing*** |
| --- | --- | --- | --- |
| *Madamesila*^60^ | 2015 | - | - |
| *Solomon*^37^ | 2016 | Phantom (CT) Vs. Patient CT | - |
| *Dancewicz*^61^ | 2017 | - | SketchUp |
| *Seoung*^30^ | 2017 | Phantom Vs. AAPM phantom | CADian 3D Ver2.0, Creator K Ver 9.6.0 |
| *Shin*^62^ | 2017 | - | SketchUp |
| *Ceh*^29^ | 2017 | Phantom (CT) Vs. Patient (CT) – digital caliper comparison | Turner MedTech, PMOD 3.2, 3D Slicer, Net Fabb |
| *Javan*^38^ | 2016 | Phantom (CT) Vs. Patient CT | OsiriX, 3D Studio Max 9.0 |
| *Kadoya*^22^ | 2017 | Phantom (CT) Vs. Patient (CT) + digital caliper comparison | ITK-SNAP |
| *Lin*^39^ | 2017 | - | - |
| *Oh*^50^ | 2017 | Phantom (CT) Vs. Patient (CT) | MATLAB |
|  |  | 3D model Vs. Patient volume difference |  |
| *Shen*^27^ | 2017 | Fidelity maps Phantoms Vs. Original CT data | Mimics, Unigraphics NX, Geomagic |
| *Craft*^49^ | 2017 | (a) Phantom (CT) Vs. Patient (CT) | TPS, OsiriX, Netfabb, Meshlab, Simply3D |
|  |  | (b) Measured Vs. Planned thickness of each slice and entire phantom |  |
| *Lee*^26^ | 2016 | Thickness of CT image Vs. Thickness of point cloud data | - |
|  |  | Phantom (CT) Vs. RANDO model |  |
| *Leng*^63^ | 2016 | Phantom (CT) Vs. Patient (CT) – brain | Medium sharp kernel, Mimics, 3 Matic |
| *Toepker*^28^ | 2013 | Area measurement True degree Vs. Measured degree | Unigraphix NXS, Reconstruction: soft-tissue kernel (B26) |
| *Hamedani*^54^ | 2018 | Phantom (CT) Vs. Patient (CT) | Cylinder (3DS Max Design), Artery tree (Creo V2), Pelvis (3-matic), Iliac artery (Mimics), & Cura |
| *Hazelaar*^55^ | 2018 | Phantom (CT) Vs. Patient (CT) | Materialise Mimics |
| *Kamomae*^51^ | 2017 | Phantom (CT) Vs. Patient (CT) | OsiriX, Shade3D, Slic3r |
| *Yoshimaru*^65^ | 2013 | MRI (Manual converted to true size and measured by digital caliper Vs. Automatic affine transformation matrix) | - |
| *Kasten*^58^ | 2016 | MRI measured, analytical, rasterized | - |
| *Wood*^36^ | 2017 |  | iSeg, Geomagic 2012 |
| *Saotome*^56^ | 2017 | Phantom Vs. Patient | MATLAB |
| *Rai*^66^ | 2018 | Tibia phantom (MRI) Vs. Patient (MRI) | MiM Maestro, Materialize Magics Version 19 |
| *Adusumilli*^45^ | 2014 | (a) Acromiohumeral distance Calipers Vs. Sonography | osiriX, Meshlab |
|  |  | (b) Phantom (US) Vs. Patient (US) |  |
| *Bucking*^33^ | 2017 | Digital model (CT) Vs. Phantom (calipers, micrometer) | Seg3D, 3D Slicer, MeshMixer, FreeCAD |
| *Fuzesi*^47^ | 2016 | (a) Row filaments (Calipers) | AutoCAD |
|  |  | (b) Phantom (CAD) Vs. Reference point |  |
| *Nikitichev*^67^ | 2016 | - |  |
| *Lai*^68^ | 2013 | Digital (CAD) model Vs. Phantom (normal photograph) | Solidworks |
| *Morais*^46^ | 2017 | Printed phantom Vs. Virtual phantom | Medical Imaging Interactive Toolkit, SolidWorks |
| *Maneas*^44^ | 2018 | - | Heart phantom (Netfabb, Meshmixer, FreeCAD, Blender), Placenta phantom (Inkspace 2016, FreeCAD) |
| *In*^69^ | 2017 | - |  |
| *Alssabbagh*^40^ | 2017 | Just mentioned digital and conventional caliper | 3Ds MAX 2013 |
| *Gallas*^20^ | 2015 | - | MITK |
| *Mitsouras*^52^ | 2017 | Phantom (MRI, CT) Vs. Digital model STL (CT) | Vitrea, Materialise |
| *Niebuhr*^42^ | 2016 | Phantom (MRI, CT) Vs. Patient (MRI, CT) | Geomagic Freeform |
| *Laing*^53^ | 2018 | Phantom (CT) Vs. Patient (CT) | 3D Slicer, MeshLab, Taubin Filter, SpaceClaim |
| *Adams*^21^ | 2016 | Phantom (CT) Vs. Patient (CT) | InVesalius |
| *Wollen-weber*^59^ | 2016 | - | - |
| *Gallivanone*^70^ | 2016 | Phantom (CT, PET, PET/CT) | - |
| *Cervino*^71^ | 2017 | Phantom (CT, PET) | SolidWorks 2014 |
| *Bieniosek*^10^ | 2015 | (a) Phantom (CT, PET) Vs. Commercial (CT, PET) | - |
| *Gear*^32^ | 2016 | Phantom (CT) Vs. Patient (MRI) | DeVide |
| *Robinson*^34^ | 2016 | Phantom (SPECT/CT) Vs. Mathematical model (volume) | - |
| *Woliner van der Weg*^72^ | 2016 | - | Mimics, MeshLab |
| *Tran-Gia*^57^ | 2016 | (a) Theoretical Vs. Measured filling volume | Slic3r |
|  |  | (b) Empty Vs. Filled weight |  |
| *Negus*^41^ | 2016 | Phantom (CT) Vs. Elliptical phantom Vs. Original | - |
| *Alqahtani*^73^ | 2017 | - | - |
| *Kiarashi*^48^ | 2015 | (a) Phantom (Mammogram)Vs. Virtual phantom (CT) | - |
|  |  | (b) Thicknesses measured by digital caliper Vs. Nominal values |  |
